# Mesenchymal stem cells-based therapy in liver diseases

**DOI:** 10.1186/s43556-022-00088-x

**Published:** 2022-07-27

**Authors:** Heng-Tong Han, Wei-Lin Jin, Xun Li

**Affiliations:** 1 The First School of Clinical Medicine, Lanzhou University, Lanzhou, 730000 P. R China; 2 Medical Frontier Innovation Research Center, The First Hospital of Lanzhou University, No. 1 West Donggang Road, Lanzhou, 730000 People’s Republic of China; 3 Department of General Surgery, The First Hospital of Lanzhou University, Lanzhou, 730000 People’s Republic of China; 4 Key Laboratory Biotherapy and Regenerative Medicine of Gansu Province, Lanzhou, 730000 People’s Republic of China

**Keywords:** Chronic liver diseases, Mesenchymal stem cells, Immune regulation, Liver regeneration, Liver immune microenvironment

## Abstract

Multiple immune cells and their products in the liver together form a complex and unique immune microenvironment, and preclinical models have demonstrated the importance of imbalances in the hepatic immune microenvironment in liver inflammatory diseases and immunocompromised liver diseases. Various immunotherapies have been attempted to modulate the hepatic immune microenvironment for the purpose of treating liver diseases. Mesenchymal stem cells (MSCs) have a comprehensive and plastic immunomodulatory capacity. On the one hand, they have been tried for the treatment of inflammatory liver diseases because of their excellent immunosuppressive capacity; On the other hand, MSCs have immune-enhancing properties in immunocompromised settings and can be modified into cellular carriers for targeted transport of immune enhancers by genetic modification, physical and chemical loading, and thus they are also used in the treatment of immunocompromised liver diseases such as chronic viral infections and hepatocellular carcinoma. In this review, we discuss the immunological basis and recent strategies of MSCs for the treatment of the aforementioned liver diseases. Specifically, we update the immune microenvironment of the liver and summarize the distinct mechanisms of immune microenvironment imbalance in inflammatory diseases and immunocompromised liver diseases, and how MSCs can fully exploit their immunotherapeutic role in liver diseases with both immune imbalance patterns.

## Introduction

Liver diseases are a major global health problem with approximately 2 million deaths per year worldwide [1]. According to the latest statistics from the World Health Organization, liver diseases accounted for 4.6% of deaths in the Asia–Pacific region in 2015, compared with 2.1% in the United States and 2.7% in European countries [2, 3]. The common types of liver diseases have changed. The prevalence of chronic hepatitis C in the United States has decreased nearly twofold compared to 20 years ago, but the prevalence of nonalcoholic fatty liver diseases (NAFLD) has been increasing in recent years [4] and has become the second most common indication for liver transplantation in the United States [5]. In addition, chronic hepatitis B virus infection remains a major contributor to liver disease-related deaths and hepatocellular carcinoma (HCC) in the Asia–Pacific region, but at the same time, NAFLD has become a notable growing liver disease in the region [3]. The progression of liver diseases caused by viral infections (e.g., hepatitis B and C), autoimmune hepatitis, alcoholic liver diseases, and NAFLD to end-stage liver failure, cirrhosis and HCC is responsible for the increased mortality from liver diseases [6]. Currently, only liver transplantation can effectively treat end-stage chronic liver diseases and save patients' lives, but the shortage of available livers for transplantation and lifelong use of immunosuppressive drugs greatly limit the clinical implementation of liver transplantation. Therefore, there is an urgent need to explore more treatment strategies for liver diseases to effectively prevent or delay the progression of chronic liver diseases to end-stage liver diseases and HCC, which is the key to treating liver diseases, reducing mortality and alleviating the medical burden.

The variety of liver diseases and the complexity of their etiology and pathogenesis pose challenges for the subsequent treatment and the development of effective therapeutic agents. In fact, the liver is a complex and unique organ that not only undertakes metabolic, biosynthetic, detoxification and excretion functions, but is also the largest immune organ in the body [7], which capable of recruiting, aggregating and activating innate and adaptive immune cells to build a diverse, dynamic and interacting hepatic immune microenvironment [8, 9]. In recent years, the use of emerging technologies such as single-cell sequencing and spatial-omics has led to an unprecedented understanding of the liver immune microenvironment landscape and its dynamics in different disease contexts. It has been found that common causes of liver diseases such as long-term viral infections, alcohol intake, intestinal microbial translocation and obesity-induced metabolic disorders can cause imbalance in the immune microenvironment of the liver, either leading to immune hyperactivation or to immunodeficiency or failure, resulting in a series of pathological changes in the liver that eventually drive these liver diseases to end-stage liver diseases such as cirrhosis and HCC [7, 8]. Corresponding immunotherapies targeting the hepatic immune microenvironment have been proposed and clinically studied, but their side effects and established efficacy need further observation. Before being formally approved for clinical treatment, these immunotherapies need to overcome the impairment of normal immune function associated with nonspecific immunosuppression and the poor improvement of clinical endpoints of interest with single-target drugs, as observed in non-alcoholic steatohepatitis (NASH) [10].

Mesenchymal stem cells (MSCs) are coming into the limelight due to their powerful immunomodulatory capacity, and their therapeutic potential in liver diseases has become a matter of interest [11–13]. Importantly, this immunomodulatory capacity of MSCs is integrative and plastic, capable of both suppressing excessive immune inflammatory responses and acting as immune enhancers by interacting with innate and adaptive immune cells in the hepatic immune microenvironment [14, 15]. Taking advantage of this property, MSCs-based immunotherapeutic strategies have been developed and investigated in inflammatory and immunocompromised liver diseases that follow different immune imbalance patterns, and their combined immunomodulatory capacity, as well as tissue repair and antifibrosis, is unmatched by systemic immunosuppressive agents or single immune targeting agents. For example, the immunosuppressive ability of MSCs has been utilized to achieve good efficacy and research results in the treatment of inflammatory liver diseases such as AIH and NASH [16, 17]. In immunocompromised liver diseases, such as chronic viral hepatitis (CVH) and HCC, this property of MSCs may help viruses and tumors to evade immune surveillance, and thus their therapeutic value has been somewhat overshadowed [18–20]. In fact, MSCs can not only interact with liver immune cells to act as direct immune enhancers, but can also be engineered to act as cellular carriers for antiviral and antitumor vaccines and carry oncolytic viruses and other types of immunomodulators to indirectly modulate the liver immune microenvironment for antiviral and antitumor purposes [21, 22]. Taking advantage of the tumor tropism and intra-tumor penetration properties of MSCs, they have been used as cellular vectors to load antitumor-related drugs to precisely target tumor tissues and then modulate the tumor microenvironment (TME) and kill tumor cells to achieve synergistic antitumor effects [22].

To this end, in this review, we present the hepatic immune microenvironment and the relevant cellular and molecular mechanisms of immune microenvironment imbalance in inflammatory and immunocompromised liver diseases to elucidate the need for immunotherapy in liver diseases and the limitations of currently developed immune-targeted drugs. Meanwhile, we focused on the immunotherapeutic potential of MSCs in liver diseases, and summarized the different immunomodulatory mechanisms and immunotherapeutic strategies of MSCs in inflammatory and immunocompromised liver diseases (including chronic viral hepatitis and HCC), with the aim of providing comprehensive and safe immunotherapeutic options for liver diseases. We conclude with a discussion of challenges in the field.

## Liver immune microenvironment

The liver receives portal and arterial blood and is an important and critical component of defense against blood-borne infections as it needs to accurately recognize, capture, and remove bacteria, viruses, and macromolecules when receiving blood-borne pathogens of enteric origin [23]. The liver must also maintain immune tolerance to antigens from nutrients or resident microorganisms to prevent causing self-injury [24]. This balance between immune activation and tolerance is essential for the normal homeostasis and function of the liver, making it the first-line immune organ [25].

**Fig. 1:**
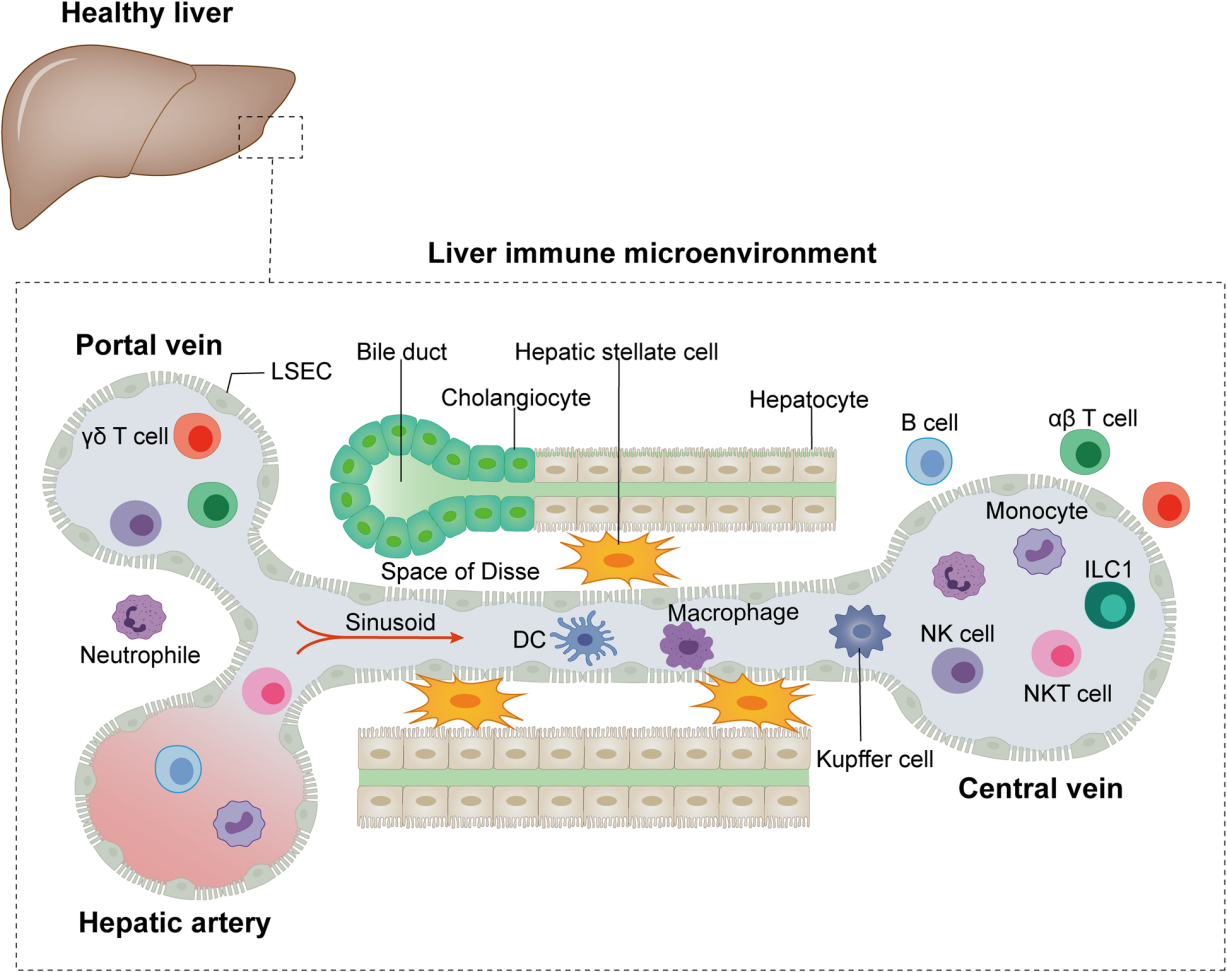
Structure and cellular composition of the immune microenvironment of the healthy liver. The hepatic lobules are the basic units that make up the tissue of the liver. The central vein is located in the center of the hepatic lobules and is surrounded by hepatocytes arranged in a radial pattern. Blood flow from the portal vein and hepatic artery converges into the central vein through the hepatic sinusoids, which are composed of endothelial cells. Bile produced by hepatocytes is released into the intestine through bile ducts composed of bile duct cells. The hepatic artery, portal vein and bile ducts are located in the confluent area between the hepatic lobules. The cells in the liver consist of resident immune cells and immune cells recruited from the circulation. The former includes Kupffer cells, DCs, LSECs, HSCs, and ILC1, etc.; the latter includes monocyte-macrophages, neutrophile, NK cells, NKT cells, αβ T cells, γδ T cells and B cells and so on. These cells are important components of the hepatic immune microenvironment and play an important role in clearing foreign bodies, initiating a rapid and controlled immune response in the face of external infections, and maintaining tolerance to autoantigens and food antigens

In the liver, a large number of innate immune cells, including Kupffer cells, dendritic cells (DCs), hepatic sinusoidal endothelial cells (LSECs), hepatic stellate cells (HSCs), natural killer (NK) cells, natural killer T (NKT) cells, γδ T cells, and recruited mononuclear macrophages, neutrophils, and adaptive immune cells, form a complex interplay network that together orchestrate the unique hepatic immune microenvironment [26–28] (Fig. 1). Here, we make the necessary update on the main hepatic immune cells that constitute the hepatic immune microenvironment and their functions.

### Macrophages

Hepatic macrophages consist mainly of liver-resident Kupffer cells and circulating monocyte-derived macrophages recruited [29]. Kupffer cells are important innate immune cells and antigen-presenting cells of the liver, derived from erythro-myeloid progenitors (EMPs) or hematopoietic stem cells-derived monocytes migrating to the fetal liver for further differentiation [30–32]. During liver injury, Kupffer cells and other hepatic parenchymal cells (e.g., hepatic stellate cells, hepatocytes) secrete chemokines such as CCL2 and many inflammatory factors, recruit monocytes to infiltrate the liver, and produce large numbers of inflammatory monocyte-derived macrophages [33–36]. Traditionally, macrophages have been assigned as inflammatory or “M1” vs. anti-inflammatory or “M2” [37], and the single-cell RNA sequencing analysis revealed the presence of two distinct populations of CD68 + macrophages in the liver, which appeared to separate into pro-inflammatory and immunomodulatory phenotypes [38]. Given the important role of macrophages, targeting macrophages has become a new strategy for the treatment of liver diseases [29].

### Dendritic Cells (DCs)

DCs play a central role in phagocytic clearance and antigen presentation by pathogens, are considered to be the most efficient antigen-presenting cells to activate naive T cells, and consist of multiple specialized isoforms [39]. The DCs family usually consists of plasmacytoid dendritic cells (pDCs) that produce type I interferon (IFN-I) and conventional DCs (cDCs), which are primarily responsible for antigen expression and immune regulation [40]. Therefore, DCs-based immunotherapies including DCs vaccines have been widely used in HCC to enhance the body’s anti-tumor immunity with a high safety profile [41].

### Liver Endothelial Cells (LSECs)

LSECs are hepatic non-substantial cells that form the sinusoidal wall, lacking basement membranes and possessing open fenestrations that allow them to efficiently regulate sinusoidal blood flow and material exchange [42]. Meanwhile, LSECs together with hepatic macrophages constitute the largest waste removal cells population in the body and are able to efficiently phagocytose and remove viral particles and waste macromolecules from the blood through endocytic receptors [43, 44]. In addition to their extraordinary scavenger functions, LSECs possess powerful immune functions, including filtration, endocytosis, antigen expression, leukocyte recruitment [45], and the ability to maintain HSCs in a quiescent state [46].

### Hepatic Stellate Cells (HSCs)

HSCs are lipid-storing cells that reside in a virtual subendothelial space between hepatocytes and LSECs (space of Disse) and also contain 50–80% of all vitamin A in the body [47]. It is widely believed that activated hematopoietic stem cells play a central role in the progression of liver fibrosis [48, 49]. A variety of immune cells and blood platelet in the microenvironment of injured/inflamed liver tissue and their extrahepatic factors can regulates the activation and apoptosis of HSCs directly or indirectly [50, 51]. Activated HSCs produce collagen fibrils and extracellular matrix components that are directly involved in liver fibrosis and are involved in the recruitment of inflammatory cells, leading to a vicious cycle between liver injury, inflammation and fibrosis in chronic liver disease and promoting the development of HCC [52].

### Hepatic lymphocytes

The liver has a large number of resident and recruited lymphocytes, including innate and adaptive immune systems. Among them, hepatic innate lymphocytes with unique characteristics, including natural killer cells (NK cells), innate lymphoid cells (ILCs), natural killer T (NKT) cells, γδ T cells, and mucosal associated invariant T (MAIT) cells, which play an important role in the maintenance of hepatic homeostasis and the progression of liver diseases and HCC [53]. In addition, αβ T cells and B cells constitute the hepatic adaptive system that exerts specific immune responses against viral and tumor antigens.

Human natural killer (NK) cells are important immune cells that can resist viral infection and clear tumor cells through direct and indirect cytotoxicity without prior sensitization [54]. NK cells have two easily distinguishable subtypes, shown as the CD56^dim^CD16 + and the CD56^bright^CD16-/ + phenotypes [55]. CD56^bright^ NK cells are strong cytokines producers (IFN-γ, TNF, GM-CSF), which are mainly involved in immune regulation but have weak cytotoxicity. However, they can also be activated by pro-inflammatory cytokines such as IL-15, thus exhibit potent antitumor responses [56]. In contrast, CD56^dim^ NK cells populations can mediate continuous killing of infected and/or malignant cells and induce apoptosis of target cells [57, 58].

Conventional αβ T cells comprise CD8 + and CD4 + T cells, both of which interact and coordinate together to establish effective hepatic adaptive immunity [25]. T cells can easily recognize and come into contact with immune cells, especially APCs, which are effectively activated and differentiated into effector and memory T cells by the combined action of antigen-presenting and co-stimulatory molecules, then exert immune effects such as antiviral and anti-tumor [59]. In liver, antigen presentation of CD4 + and CD8 + T cells is usually accomplished by professional APCs called DCs and non- professional APCs, including Kupffer cells, B cells, LSECs, HSCs and even liver cells [60, 61]. Therefore, a new era of liver immunotherapy has been opened by understanding the functional biology of hepatic T cells and the application of T cells-based immunotherapy.

In conclusion, the composition of the hepatic immune microenvironment is complex and dynamic, and its true nature has not yet been fully revealed, even with the help of single-cell sequencing and spatial-omics technologies. Maintenance of hepatic immune tolerance and immune activation against pathogens requires the normal and efficient functioning of these liver-resident and recruited innate and adaptive immune cells. However, in the context of liver diseases, profound and complex changes in the hepatic immune microenvironment occur, which have a dramatic impact on the occurrence and development of the liver diseases [62, 63].

## Imbalance of liver immune microenvironment and liver diseases

**Fig. 2:**
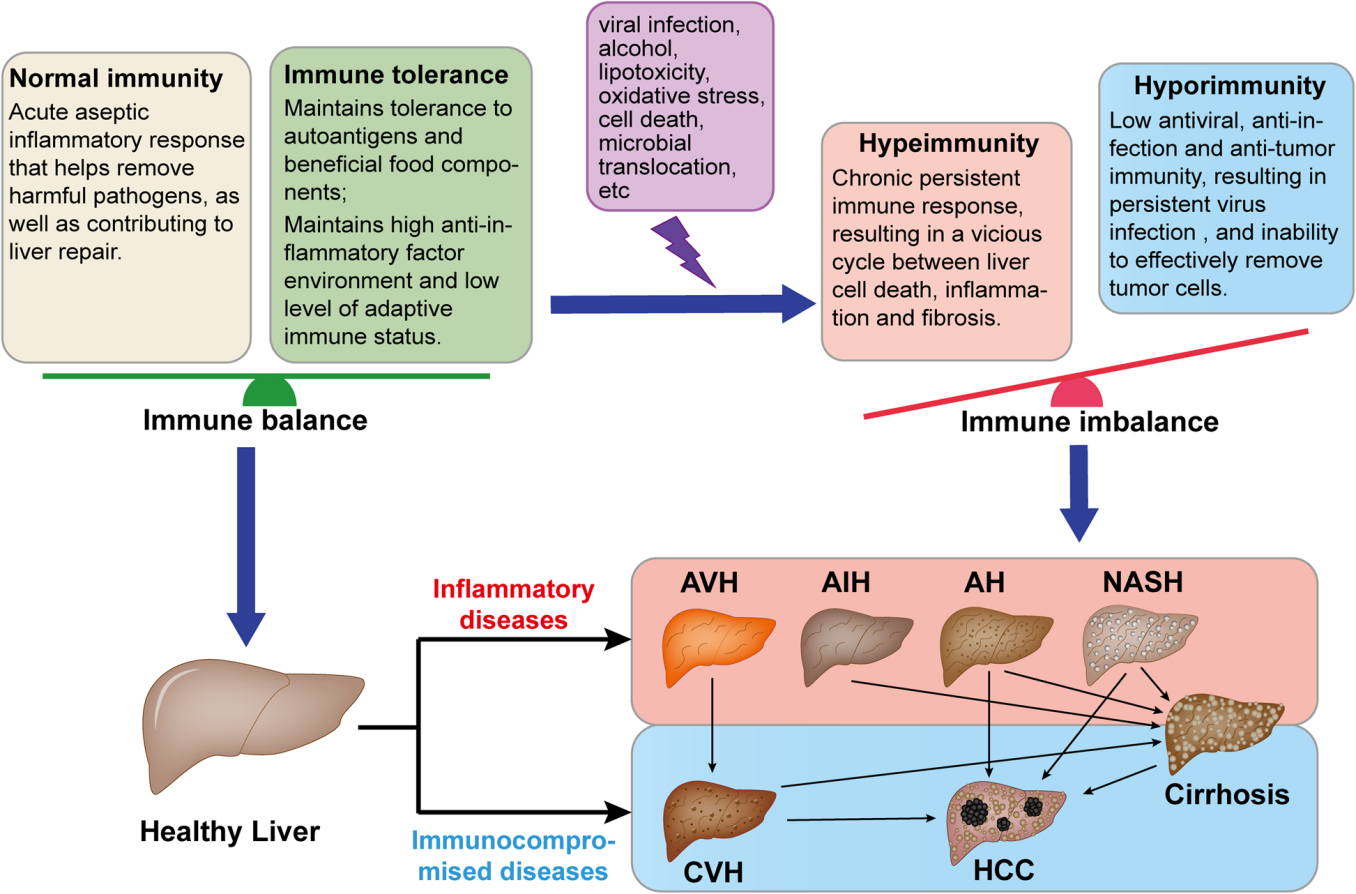
The relationship between imbalance in the hepatic immune microenvironment and liver diseases. Normal hepatic immunity and immune tolerance together maintain the balance of the immune microenvironment. The immune microenvironment is disrupted under the stimulation of multiple external factors. On the one hand, excessive immune activation leads to the development and progression of inflammatory liver diseases, including AIH, AVH, AH, NASH, and cirrhosis. Among them, cirrhosis is characterized by the coexistence of inflammation and immune deficiency. On the other hand, low antiviral and antitumor immunity leads to the development and progression of immunocompromised liver diseases, including CVH and HCC. Without effective treatment, these chronic liver diseases will gradually develop into cirrhosis and then transition to HCC, or can develop directly into HCC

Liver diseases have a complex pathogenesis and a high degree of heterogeneity, and the genetic factors and diverse environmental triggers of these diseases are not fully known. However, it is well known that stimulation by external factors, such as viruses, alcohol, hepatic lipids and intestinal microbial metabolites, can cause abnormalities in hepatic immune cells and recruitment of circulating cells, leading to a disruption of the balance of the hepatic immune microenvironment. This immune imbalance can lead to a series of immunopathological alterations that can result in liver disease [7, 8] (Fig. 2). Specifically, immune imbalance in liver diseases is characterized by two distinct pathologies: in the first, excessive immunity in the absence of infection leads to the development of inflammatory liver diseases (e.g. autoimmune hepatitis (AIH) [64, 65], acute viral hepatitis (AVH) [66, 67], alcoholic hepatitis (AH) [68, 69], non-alcoholic steatohepatitis (NASH) [70, 71], cirrhosis [72, 73]). In the second, failure to initiate an effective immune response when needed, i.e., low/insufficient hepatic antiviral and antitumor immunity, may lead to the formation of chronic viral infections or failure to clear HCC cells [74–76]. In this section, we will discuss inflammatory liver diseases and immunocompromised liver diseases separately, summarizing the specific immunopathogenesis of each liver disease. A comprehensive understanding of the immunopathogenesis and immunopathological manifestations of these liver diseases will facilitate the development of immunotherapeutic strategies with potential applications.

### Imbalanced liver immune microenvironment in inflammatory liver diseases

#### Autoimmune hepatitis: T cells-centered autoimmune injury

AIH is an autoimmune response-mediated inflammation against hepatocytes, characterized by elevated serum transaminases, positive serum autoantibodies, hyper-immunoglobulin G and/or γ-globulinemia, and histological manifestations of interface hepatitis [64]. The current view is that in genetically susceptible individuals, environmental pathogens and certain chemical agents trigger a T cells-mediated immune response against liver autoantigens through a molecular mimicry mechanism [77]. Importantly, even if the interaction between external pathogenic factors and autoimmune T cells ceases, T cells-mediated liver injury as well as inflammation persist and are mutually causal, leading to the progression of AIH to cirrhosis and liver failure in severe cases [64, 78].

**Fig. 3:**
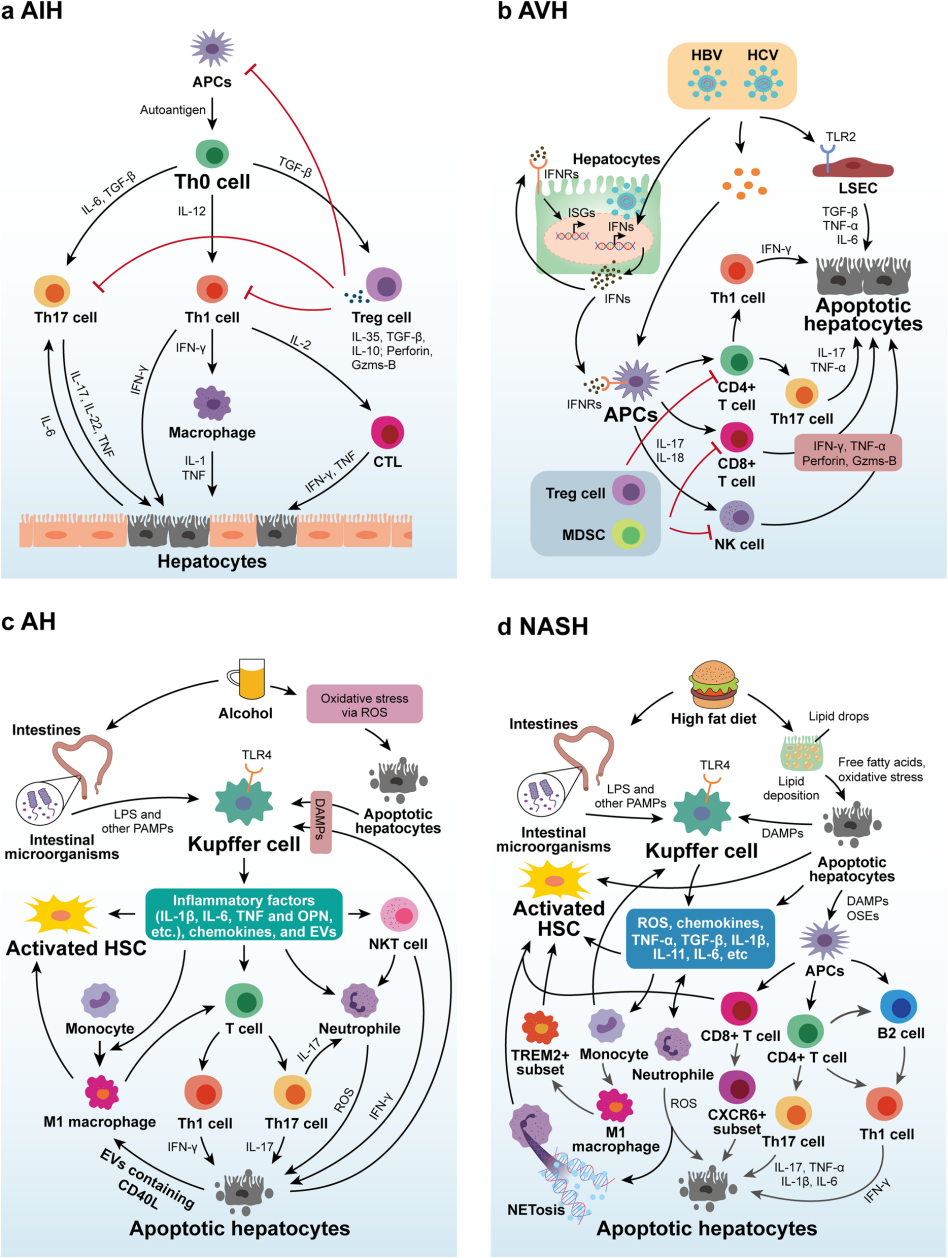
Cellular and molecular mechanisms behind the imbalance of the immune microenvironment in liver diseases. Inflammatory liver diseases including (**a**) AIH, (**b**) AVH, (**c**) AH and (**d**) NASH share the same pathological features and manifest as hepatic inflammation, hepatocyte death and liver fibrosis mediated by innate and adaptive immune cells

Thus, T cells-mediated killing of self-liver cells is a major feature of the altered immune microenvironment in AIH, including the production of cytotoxic CD8 + T cells (CTLs), as well as impaired immune regulation of CD4 + T cells subsets and Treg cells [79] (Fig. 3a). Initially, autoantigens are mishandled by antigen-presenting cells and presented to naive CD4 + T helper (Th0) cells. Th0 cells are then activated in response to appropriate costimulatory signals and mature and differentiate into different T helper subsets such as Th1, Th2, and Th17 cells in a different cytokine environment [64]. Th1 cells produce cytokines such as IL-2 and interferon-γ (IFN-γ) that promote the activation of CTLs, thus exert their cytotoxic effects [80]. IFN-γ also induces the activation of monocytes, DCs and NK cells, promoting autoimmune responses and liver injury [81, 82]. Th2 cells induce B cells to mature into plasma cells by secreting cytokines, which in turn secrete autoantibodies to attack normal hepatocytes in an antibody-dependent cytotoxic (ADCC) and complement-dependent manner causing hepatocyte death [83]. For example, multiple autoantibodies can be detected in AIH patients and the titer levels of these autoantibodies are positively correlated with the degree of disease activity [84, 85]. Th17 cells are also involved in the pathogenesis of AIH, which is associated with their production of cytokines such as IL-17, IL-22 and TNF [86, 87]. IL-17 can also induce IL-6 expression in hepatocytes by stimulating the MAPK signaling pathway, which in turn further stimulates Th17 cells, forming a positive feedback loop [88]. Nevertheless, the role of Th17 cells in AIH is still under investigation [89].

In the course of T cells-mediated autoimmune responses against hepatocytes, in addition to alterations in the immune effects of T cells subsets, alterations in T cells immune checkpoints are also included. Detection in liver tissue of AIH patients not only observed aggregation of CD4 + and CD8 + T cells overexpressed by immune checkpoint PD-1 and 4-1BB, but also found that PD-1 + CD8 + T cells were strongly associated with disease activity and degree of liver injury in AIH patients rather than PD-1 + CD4 + T cells [90]. In addition, a study isolated circulating SLA-specific CD4 + T cells. The study found that the autoreactive T cells receptor clone type was limited to memory PD-1 + CXCR5- CD4 + T cells, and the co-expression levels of PD-1 and CD38 in T cells reflect the degree of disease activity of AIH [91]. Zhao et al. showed that by using immunotoxic complexes that block PD-1, a reduction in the number of PD-1-positive cells, total T lymphocytes, and especially autoreactive T cells, could be observed in different mouse models of autoimmune disease without negatively affecting normal adaptive immunity [92].

Treg cells are immunosuppressive cells that suppress CD4 + T cells and CTLs-mediated autoimmune responses by secreting immunosuppressive cytokines such as IL-10, TGF-β and IL-35, as well as granzyme and perforin [93]. Studies on whether Treg cell function is abnormal are not uniform [94–96]. However, most published data suggest that Treg cells are defective in number and function in AIH [97–99]. Treg cells are significantly impaired in number during active phase of the disease, while the reduced sensitivity of Treg cells to IL-2 leads to a defect in IL-10 production [100]. The controversial nature of the aforementioned studies is partly due to the heterogeneity of Treg cells and the differences in defining Treg cells markers. Indeed, studies relying on CD25 expression tend to find a decrease in Treg cells, while studies relying on foxp3 expression show an increase in Treg cells [101, 102]. A phase I clinical trial to evaluate the efficacy and safety of Treg cells for the treatment of AIH is underway (NCT02704338).

In summary, AIH is an adaptive immune cells-mediated autoimmune liver disease with a core of T cells and has been extensively studied. Less research has been done on the innate immune system, but some evidence suggests that gut microbes can activate macrophages and may be involved in the pathogenesis of AIH [103]. Given the complexity of the hepatic immune microenvironment, it is believed that the hepatic immune landscape of AIH will be further refined in the future to better guide clinically accurate immunosuppressive therapy.

#### Acute viral hepatitis: antiviral immune-mediated liver injury

Hepatitis B and C viruses belong to hepatotropic viruses, which can infect hepatocytes and are the main types of viruses that cause AVH in clinical practice [104]. Symptoms of acute hepatitis C virus (HCV) infection are usually subclinical, whereas acute hepatitis B virus (HBV) infection tends to result in symptomatic hepatitis in adults, with patients' clinical presentation ranging from asymptomatic to fulminant liver failure [105, 106]. Data show that 23.1% of patients with acute viral hepatitis B-related acute liver failure eventually die [107].

As an immune organ, cells in the liver immune microenvironment can rapidly recognize and initiate their own antiviral immune response at the early stages of viral invasion and work closely with each other to clear the infected virus [108, 109]. Briefly, once activated, the hepatic antiviral immune system first exerts its antiviral immune action by releasing interferon to act on antigen-presenting cells and activating adaptive immune cells, and awakens the immune response of IFNs in uninfected hepatocytes to defend against viral attack [110]. HBV has been considered a recessive virus because it has rarely been observed to induce an obvious immune response to IFNs [111]. However, several studies have also shown that HBV can induce such intracellular immune responses under specific experimental conditions s [112–114]. A recent study showed that MX2 is an important IFN-α inducer that effectively reduces HBV-RNA levels and inhibits HBV replication by indirectly impairing the formation of cccDNA [115]. Unlike HBV, HCV induces high levels of ISGs expression while often evading the innate immune response through elaborate strategies and exhibiting a propensity for chronic infection [116].

A strong and rapidly responsive innate and especially adaptive immune response is necessary to control acute viral infections, but also consequently induces immunopathological liver damage and inflammation [117], which in severe cases will trigger acute liver failure and lead to death. The immunopathogenesis of AVH, although still not fully clarified, has come a long way [118] (Fig. 3b). The central role of virus-specific CD8 + T cells in the clearance of acute viral infection and virus-associated liver injury is now well established [119, 120]. For example, CD8 + T cells and NK cells clear infected cells by secreting antiviral cytokines (e.g., IFN-γ) and with perforin-dependent cytotoxic effects, but this leads to indiscriminate liver injury [121, 122]. By constructing a mouse model of AVH-induced liver failure, investigators found that virus-specific CD8 + T cells not only induced liver injury in a perforin-dependent manner, but their mediated elimination of LSECs led to loss of endothelial integrity of the liver sinusoids and severely impaired sinusoidal perfusion, which indirectly led to hepatocyte death [123]. In addition, when HBV-specific CD8 + T cells fail to control viral replication, they can also recruit non-viral-specific T cells, which leads to further liver injury [124]. In addition to CD8 + T cells, both HBV and HCV infection can promote the recruitment of hepatic Th17 cells, which in turn can exacerbate liver injury, inflammation and even fibrosis during viral infection through paracrine effects [125, 126]. Excessive Th1 and Th17 cytotoxic responses, as well as secreted IL-17 triggering IL-8-mediated recruitment of hepatic neutrophils, have been shown to be associated with the development of HBV-associated liver injury and inflammation [127].

Treg cells and MDSCs are able to inhibit these cells-mediated pathological injury to some extent and do not affect their normal antiviral immune capacity. For example, foxp3 + Treg cells have been shown to protect the liver from immune injury in the early stages of acute HBV infection without affecting the proliferation of HBV-specific CD8 + T cells and memory T cells [128]. In addition, CD4 + CD25 + Tregs directly inhibit NK cell-mediated hepatotoxicity by interacting with NK cells through mTGF-β and OX40/OX40L in a cell-contact manner [129]. Treg cells also control the recruitment of innate immune cells such as macrophages and dendritic cells, thereby reducing liver inflammation, although this leads to some degree of HBV clearance delayed [128]. Another type of suppressor cells, myeloid-derived suppressor cells (MDSCs), have been shown to inhibit T- and NK-cells-mediated liver injury in AVH by producing arginase [130–132].

In conclusion, the hepatic antiviral immune response leads to the death of virally infected hepatocytes, but also damages uninfected cells, and the liver damage itself induces further inflammation exacerbating the liver damage. In addition to the cytotoxic cells-mediated liver injury described above, recent studies have revealed an emerging role for LSECs in liver injury in AVH. In the mouse hepatitis 3 virus (MHV3)-induced AVH model, LSECs undergo a shift from an anti-inflammatory to a pro-inflammatory phenotype characterized by the release of the pro-inflammatory factors TGF-β, IL-6, and TNF-α, and reduced IL-10 secretion [133]. This phenotypic shift is associated with virus-induced activation of TLR2 signaling and also corresponds to the severity of hepatitis [133]. Thus, moderate immunity and inflammation are necessary to clear the virus, while excessive and uncontrolled immune responses are detrimental. How to strike a delicate balance between antiviral and anti-damage to maximize the therapeutic effect in patients with AVH is a question that deserves further investigation.

#### Alcoholic hepatitis: inflammatory dysregulation due to alcohol exposure

Alcohol-related liver disease (ALD) is a chronic liver disease caused by long-term, high-frequency alcohol intake with poor treatment response, prognosis and survival [134]. Alcoholic hepatitis (AH) is a clinical form of ALD characterized by acute alcohol-induced liver injury as a pathological manifestation [135]. In particular, the clinical prognosis of severe AH is poor, with 40% of patients with severe AH dying within 6 months of the onset of clinical symptoms [136]. Recent studies have shown that hepatic immune cells and the gut-liver axis play a key role in the development of alcohol-induced hepatocellular injury, liver inflammation and liver fibrosis [137–139].

Alcohol is sufficient to cause inflammatory liver damage directly or indirectly [140]. In the presence of a sustained increase in ethanol, the accumulation of its toxic metabolite acetaldehyde in the liver increases causing oxidative stress, which generates reactive oxygen species and induces endoplasmic reticulum stress and mitochondrial dysfunction, ultimately leading to hepatocyte apoptosis and dysfunction of various immune cells in the liver [141, 142].

In addition to the direct effects caused by alcohol, activation of Kupffer cells by intestinal microbial metabolites (e.g., LPS) that break the intestinal mucosal barrier under the damaging effects of alcohol is thought to be the initiating link in triggering liver inflammation in AH [143, 144] (Fig. 3c). PAMPs such as bacterial endotoxin and LPS are recognized by TLR4 on the surface of Kupffer cells, and then activated Kupffer cells trigger the maturation of IL-1β via the inflammatory vesicle pathway[145] and secrete other active factors such as TNF, chemokines, acute phase response proteins and extracellular vesicles (EVs) [143, 144], activating liver-resident immune cells and recruit neutrophils and lymphocytes, which are involved in shaping the hepatic pathological features of AH [146]. Importantly, TLR4 receptors on Kupffer cells further recognize damage-associated molecular patterns (DAMPs) released after hepatocyte injury, creating a vicious cycle between liver injury, inflammation, and fibrosis [143]. In addition, alcohol can transform hepatic macrophages into an M1 phenotype characterized by increased production of inflammatory cytokines and ROS [147, 148], which may be associated with NOTCH1 signaling-mediated metabolic reprogramming [149]. Early evidence from studies on ALD mouse models and ALD patients suggests that pathogenic macrophage subpopulations can be successfully translated into new options for disease treatment [150]. Recent study has demonstrated that hepatocytes respond to alcohol exposure in a caspase-dependent manner by releasing EVs containing CD40L, which in turn leads to the activation of macrophages [151].

One of the remarkable hepatic pathological features of AH patients is neutrophil infiltration and is associated with patient survival [152]. These neutrophils not only participate in inflammatory liver injury by producing ROS, but also exhibit insufficient phagocytic and bactericidal activity to effectively control infection, hence the high rate of infection and mortality in patients with advanced AH [153]. The presence of defects in the IL-33/ST2 pathway in patients with severe AH has been shown to be associated with a reduced ability of neutrophils to migrate, leading to a higher chance of infection in patients [154].

T lymphocytes are widely present in the liver of ALD patients and are significantly associated with liver inflammation, sclerosis and Kupffer cells abnormalities [155]. Identification of disease-associated differential TCRs by high-throughput assays provides evidence of a unique antigen pool present in AH to activate bystander and antigen-specific T cells responses [156]. The product of lipid peroxidation originated from alcohol consumption, malondialdehyde (MDA), 4-hydroxynonenal (HNE), can be used as a neoantigen to activate T cells and B cells immune responses [157]. Indeed, Each T cells subset plays a different role in the pathogenesis of ALD by producing characteristic cytokine profiles [158]. Th1 cells mediate specific cellular responses to alcohol dehydrogenase (ADH) in AH mainly through secretion of IFN-γ [159, 160]. Th17 cells not only recruit neutrophils by secreting IL-17 [161], but also contribute to liver repair by producing IL-22 through STAT3 activation [162]. More importantly, using an alcohol-induced HCC model with global IL-17A gene defect, it was found that drug blockade of IL-17A/Th17 cells was consistent with IL-17A knockdown and could effectively inhibit the progression of HCC in alcohol-fed mice [163].

NKT cells are a subpopulation of T cells with two phenotypes, pro-inflammatory type I NKT cells and anti-inflammatory type II NKT cells. IL-1β from Kupffer cells after alcohol exposure is able to recruit and activate hepatic pro-inflammatory type I NKT cells [164], which subsequently induce neutrophil infiltration into the liver [165]. Furthermore, in a chronic AH model, type I NKT cells show high expression of Fas and FasL and secrete IFN-γ, suggesting that they can directly cause liver injury [166].

In summary, AH is a severe clinical stage of ALD in which continuous exposure to alcohol causes liver damage, inflammation, and subsequent liver fibrosis in both direct and indirect ways. The study of its immunopathogenesis has made great breakthroughs in recent years. Moreover, infection is an important cause of poor prognosis in AH, especially in severe AH, suggesting that activated immune cells may have functional abnormalities, as manifested in neutrophils. Whether similar properties exist in other immune cells, especially T cells, needs to be further investigated and is crucial to prevent the progression of AH to HCC. Therefore, immunotherapy is a promising future therapeutic strategy for AH, and in addition to effectively suppressing inflammation, the ability of the liver to control infection also needs to be preserved.

#### Non-alcoholic fatty hepatitis: inflammation-centered metabolic syndrome

Non-alcoholic fatty liver disease (NAFLD) is a heterogeneous disease that includes a range of hepatic manifestations starting with hepatic steatosis, liver injury and inflammation and progressing to cirrhosis and even hepatocellular carcinoma in NASH [167]. NASH is a clinically severe form of NAFLD, the incidence of which has increased significantly worldwide in recent years and in 2019 has become the second largest and fastest growing indication for liver transplantation in the United States [5]. Innovative therapies have been developed in an attempt to treat this growing chronic disease, but there are currently no approved clinical therapies [10]. Several studies have already suggested that factors from adipose tissue or the gut (e.g., LPS and other endotoxins, bile acids, free fatty acids), as well as insulin resistance-driven lipid accumulation and hepatic oxidative stress within the liver, together initiate intracellular stress pathways that induce hepatocyte injury and activation of inflammatory cells in NASH [70, 168–170]. It is clear that inflammation has become a central event in the progression of NASH [170], and inflammation and liver injury promote each other, creating a vicious cycle of liver injury, inflammation and fibrosis in NASH (Fig. 3d).

Innate immune cells are considered to be important players in liver inflammation in NSAH. NASH involves activation of Kupffer cells and recruitment of leukocytes, such as neutrophils, monocytes, and NK cells. The aforementioned cells, as well as hepatocytes [171], produce biokines such as cytokines, chemokines, nitric oxide and reactive oxygen species, which stimulate liver inflammation, hepatocyte steatosis, apoptosis and necrosis as well as induce fibrosis in NASH [172–174]. Among them, cytokines such as TNF-α [175, 176], TGF-β [177], IL-11 [178] and IL-1 [179] are essential for pathological characterization of NASH.

The inflammatory response triggered by Kupffer cells together with apoptotic hepatocytes becomes an early event that drives the liver from steatosis to steatohepatitis. Pro-inflammatory cytokines, LPS or other PAMPs derived from intestinal bacteria, DAMPs released from apoptotic hepatocytes and lipid metabolites (e.g., free fatty acids) all contribute to the activation of Kupffer cells and subsequent recruitment of circulating monocytes to the liver. Several studies have revealed the fate, ecological niche and regulatory landscape of liver tissue-resident and recruited macrophage populations in NASH by single-cell sequencing. These studies found that hepatic Kupffer cells were actually reduced, and that infiltrating monocytes had at least two fates: monocyte-derived Kupffer cells (MoKC), which replenished the depleted of Kupffer cells pool in liver, and monocyte-derived lipid-associated macrophages (LAM) or scar-associated macrophages (SAM) with the expression of CD9, TREM2 and osteopontin, which show differences in lipid and inflammatory genes and an association with fibrotic ecotopes [180–182]. The central role of macrophages in the pathogenesis of NASH makes them a potential target for NASH therapy [183].

In NASH patients, neutrophils are recruited to infiltrate the periportal vein and are a source of IL-17 in NASH [184, 185]. In a mouse model of NAFLD, depletion of neutrophils by antibodies suppressed metabolic dysregulation, liver inflammation and fibrosis in mice [186]. Liver-infiltrating neutrophils mediate the inflammatory response between neutrophils and other inflammatory cells such as macrophages through the production of ROS, the release of a large number of granular proteins (e.g., myeloperoxidase, neutrophil elastase, etc.) [187–189]. In addition, neutrophils release a structure called neutrophil extracellular traps (NETs) during a self-induced death process called NETosis, which is thought to contribute to the development of inflammation and liver fibrosis in NASH [187]. In mice, neutrophil infiltration and NETosis can promote the progression of NASH to hepatocellular carcinoma [190].

There is growing evidence that adaptive immunity is an additional factor promoting liver inflammation. The factors that activate CD4 + T cells and the mechanisms of immune effects are unclear and remain to be investigated. Early evidence suggests that DCs and other APCs provide OSEs to CD4 + T helper cells, leading to activation and polarization of CD4 + T cells [191–193]. It has also been shown that OX40 expression in CD4 + T cells mediates infiltration and differentiation of hepatic CD4 + T cells to Th1 cells and correlates with liver inflammation and disease severity [194]. B2 cells can influence the polarization of T cells. Studies have shown that B2 cells are activated earlier than T cells and that selective deprivation of B2 cells prevents maturation of plasma cells and polarization of CD4 + T cells to Th1 cells and effectively ameliorates steatohepatitis [195]. In addition, a CXCR3 + Th17 cell (ih Th17 cells) has recently been identified in NASH, a subpopulation of pro-inflammatory Th17 cells whose cellular metabolism is characterized by increased glycolysis and exerts its pathogenic potential through the production of IL-17A, IFN-γ and TNF-α [196].

CD8 + T cells were found to regulate inflammation and liver injury in obesity-associated NASH and activate hepatic stellate cells to promote the development of disease fibrosis, in contrast to not observed in lean NASH models [197]. Recent studies have found that fatty liver microenvironment-induced autoaggressive CXCR6 + CD8 + T cell subsets, unlike antigen-specific CD8 + T cells, can promote liver injury and conversion of NASH to HCC by secreting pro-inflammatory cytokines and directly killing hepatocytes in a FASL-dependent and TNF-dependent manner [198]. In addition, accumulation of PD1 + CD8 + T cells was observed in the liver of NASH patients, which may lead to tissue damage and impaired immune monitoring and reduce the responsiveness of NASH-HCC patients to PD-1/PD-L1 immunotherapy [199].

In summary, the idea that NASH is centered on inflammatory events has been widely accepted, and the immune cells involved in inflammatory events involve not only innate immune cells but also adaptive immune cells. Although the intra- and extra-hepatic factors that initiate immune cells, and the changing patterns of phenotype, function, and cellular metabolism of these immune cells remain somewhat elusive, further understanding of the immunopathogenesis of NASH could help in the development of innovative target drugs.

#### Cirrhosis: coexistence of inflammation-mediated fibrosis and immunodeficiency

**Fig. 4:**
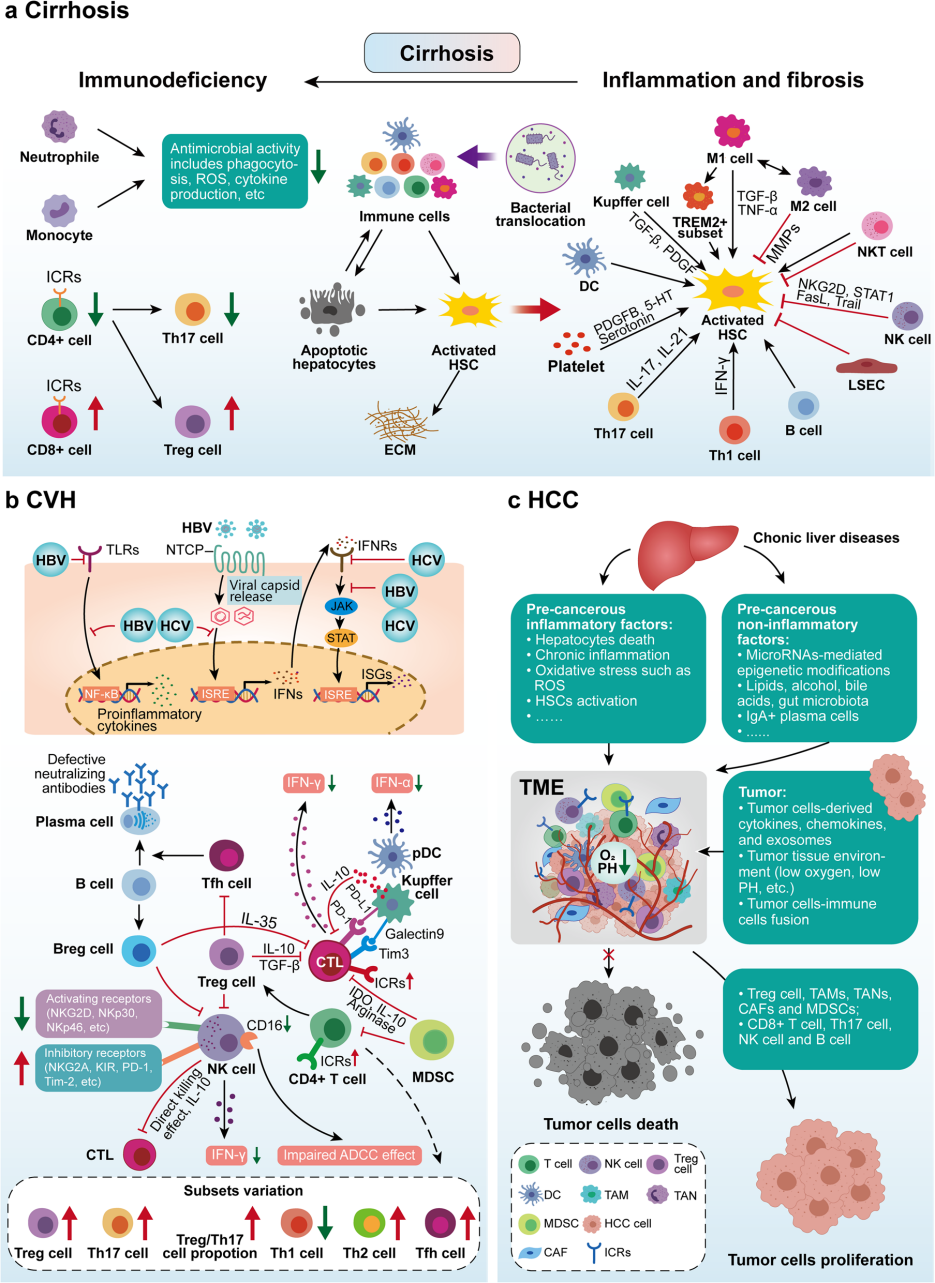
Cellular and molecular mechanisms behind the imbalance of the immune microenvironment in liver diseases. The early stages of (**a**) cirrhosis are characterized by a vicious cycle of liver inflammation, liver damage and fibrosis, whereas the advanced stages are characterized by immunodeficiency. Immunocompromised liver diseases include (**b**) CVH and (**c**) HCC, which have immunocompromised hepatic innate and adaptive immune cells resulting in impaired immune surveillance against viruses and tumors

Fibrosis is a highly conserved response to liver injury, and liver fibrosis and its end-stage cirrhosis are the ultimate common pathway in almost all chronic liver diseases, the development of which is observed in patients with chronic viral hepatitis, NAFLD, ALD, cholestasis and autoimmune liver diseases [200]. Liver fibrosis implies an excessive accumulation of extracellular matrix (ECM) caused by the activation of hepatic stellate cells and their production of collagen, etc., as well as a failure in the regulation of ECM degradation. More importantly, the formation of ECM and aberrations in the liver regeneration process lead to abnormalities in liver structure and function, eventually leading to cirrhosis and its end-stage liver failure [201]. Adequate studies have shown that liver fibrosis can be reversed to some extent by reducing liver damage and controlling inflammation, but there is still no effective and applicable treatment for liver fibrosis itself [202]. The imbalance of the immune microenvironment in cirrhosis is unique and is characterized by the coexistence of inflammation and immune deficiency [73, 203] (Fig. 4a).

##### Inflammation and fibrosis

Liver fibrosis is a multicellular response in which activated HSCs differentiate into myofibroblasts that act as major effector cells and produce ECM [48, 204]. The activation of HSCs is complex and plastic, resident and recruited cells in the hepatic immune microenvironment and platelets regulate the activation of HSCs, and these new findings also inform the development of immunotherapeutic strategies against liver fibrosis [73, 205–207].

Hepatic macrophages are considered to be the key cells in the development and regression of liver fibrosis [208, 209]. Inflammatory cytokines and chemokines, such as TNF-α, IL-6 and IL-1α, as well as CCL2, produced during the hepatic damaging inflammatory response, promote the activation of Kupffer cells and the recruitment and differentiation of circulating monocytes, and then activate HSCs to cause collagen production [210]. However, hepatic macrophages are a very heterogeneous population of immune cells, and different phenotypes of macrophages play opposite roles in inflammation and liver fibrosis [211]. For example, infiltrating Ly6C + monocyte-derived macrophages are associated with chronic inflammation and fibrosis. During fibrosis regression, monocyte-derived cells differentiate into Ly6C (Ly6C, Gr1) low-expressing “restorative” macrophages and promote the regression of injury [212]. Thus, macrophages are a cellular regulator of liver fibrosis deposition and resolution [213].

TREM-1 is an activating receptor expressed on the surface of several innate immune cells and is responsible for inflammatory regulation and inflammatory signaling [214]. It has been demonstrated that the TREM-1 pathway on Kupffer cells plays a crucial role in liver inflammation and fibrosis in a mouse model of fibrosis by promoting the infiltration of inflammatory macrophages and the activation of HSCs. Deletion of TREM-1 alleviated liver injury, inflammatory cell infiltration and fibrosis in mice [215]. The recently identified monocyte-derived TREM2 + CD9 + macrophages subset is thought to be a specific macrophage subpopulation that promotes NASH fibrosis, unlike monocyte-derived Kupffer cells [180–182]. In a first-in-human phase 1 trial of autologous macrophages for cirrhosis, patients infused at different doses showed no adverse effects, meeting the primary endpoints of safety and feasibility [216]. However, we must realize that the study of the interaction between hepatic immune cells and hepatic stellate cells must rely on reliable ex vivo experimental models to better model the complex ecological niche of HSCs. The concrete immune mechanisms of cirrhosis still need to be investigated in depth.

##### Immunodeficiency in cirrhosis

Cirrhosis is not only a pathological feature manifested by inflammation, fibrosis, tissue repair and vascular remodeling in the liver, but also a clinical syndrome called cirrhosis-associated immune dysfunction (CAID) manifested by increased intestinal microbial translocation and the coexistence of systemic persistent inflammation and immune deficiency [73, 203]. As the disease progresses and worsens, in patients with cirrhosis in end-stage liver failure, the “pro-inflammatory” systemic inflammatory phenotype transforms into an “immunodeficient” systemic inflammatory phenotype, leading to immune paralysis. This undoubtedly increases the risk of bacterial infection and the persistence of systemic inflammatory response, and leading to patient deterioration and multi-organ failure [73].

The immunodeficient phase of CAID, in which innate and adaptive immune cells and their functions are extensively damaged [205, 217]. The antimicrobial activity of innate immune cells such as circulating neutrophils and monocytes is severely compromised, leading to disease progression [205, 218]. In addition, earlier than the onset of ACLF, adaptive immune cells are impaired in cirrhosis, as evidenced by a decrease in Th0 cells and effector T (Teff) cells [217]. In detail, the frequency of Th1 cells was significantly higher, the frequency of Th17 cells was lower, and the relative number of Treg cells was increased in patients with acute decompensated cirrhosis/ACLF; meanwhile, the proportion of CD8 + T cells was significantly higher in all stages of cirrhosis [217]. Importantly, changes in CD4 + and CD8 + T cells are not only reflected in numbers and subpopulations, but co-stimulatory molecules and immune checkpoints on these cells are upregulated and the production of pro-inflammatory cytokines is significantly reduced [217], and these changes may increase the risk of infection and ACLF in patients with cirrhosis.

To summarize, cirrhosis is the common end-stage of several liver diseases, where inflammation-driven fibrosis is the main pathological feature and a vicious cycle between hepatocyte injury-inflammation-fibrosis is evident behind its pathological progression. From an immunological perspective, inflammation and immunodeficiency coexist in cirrhosis, two often overlapping entities, and research on their specific immunological features is just beginning. How exactly does the landscape of the immune microenvironment change as cirrhosis progresses to HCC? What are the factors influencing it and through which pathways? These remain to be further explored.

### Imbalanced liver immune microenvironment in immunocompromised liver diseases

#### Chronic viral hepatitis: low antiviral immunity

HBV and HCV remain the major pathogen types causing chronic viral hepatitis worldwide [219, 220]. HBV and HCV are both hepatotropic viruses, but different virology and immunology determine their different ways and manifestations of chronic infection [221]. Most adults who are infected with HBV tend to present with self-limited infection, which leads to persistent chronic infection in a subset of immunocompromised or deficient adults and in children with vertical transmission through mother-to-child transmission [117, 222]. In contrast, HCV tends to progress to chronic infection, and its clinical manifestations in the untreated setting are marked and continuously progressive [116].

The immunological profile of acute and chronic viral infections is different [221], with the former focusing more on hepatic immune-mediated damage and the latter on the antiviral immunodeficiency behind persistent viral infection. Both HBV and HCV have evolved mechanisms to evade the body’s antiviral immunity during chronic infection, including evasion of recognition, prevention of interferon production, expansion of immunosuppressive cells, and upregulation of cytotoxic cytosolic immune checkpoint receptors (ICRs), resulting in persistent infection in liver tissues and high blood loads of virus [223, 224] (Fig. 4b).

#### Interferons

Both HBV and HCV have evolved complex mechanisms to evade host intrinsic immunity, including evasion of complement and antibody recognition and killing, evasion of recognition of intracellular and extracellular PRRs, inhibition of cellular PRRs downstream signaling pathways (IRF, NK-κB, and JAK/STAT signaling pathways), and thus evasion of interferon-mediated antiviral effects [225]. In addition, some IFNs isoforms may have a negative effect of mediating viral immune escape. For example, a recent study demonstrated that IFNλ4-induced endoplasmic reticulum stress impairs HCV antigen processing and presentation to CD8 + T cells, which directly leads to attenuated HCV-specific T cells responses [226]. Besides, interferon-induced transmembrane proteins (IFITMs) are innate effector proteins that may exert significant selective pressure on HCV during the acute phase of infection, leading to viral evasion of antibody-mediated neutralization responses [227].

#### Innate cellular immunity

##### DC cells

The role of DCs in CVH needs further clarification. Several studies have shown that in chronic HBV infection, HBV is able to inhibit TLR9-mediated IFN-α production in pDCs cells [228–230], and the number of pDCs and TLR9 expression are inversely correlated with serum HBV viral load [230]. HBsAg also inhibits IFN-α production by pDCs by inducing TNF-α and IL-10 production in monocytes [231]. Similar to HBV, HCV core protein leads to TNF-α and IL-10 production through activation of monocyte TLR2 signaling, which leads to increased apoptosis in pDCs and their impaired ability to secrete IFN-α [232].

##### Kupffer cells

HBV and HCV infection can impair the antiviral activity of Kupffer cells by interfering with PRRs receptor-mediated signaling, inhibiting the release of pro-inflammatory cytokines such as TNF-α, and increasing the production of anti-inflammatory factors [233–235]. Kupffer cells-mediated viral immune escape is at least partially related to their induction of CTLs depletion. HBV and HCV infection can upregulate the expression of the inhibitory ligands PD-L1 and galactose lectin-9 on the surface of Kupffer cells and induce the failure of CTLs by binding to the corresponding inhibitory receptors PD-1 and Tim-3 on the surface of CD8 + T cells [233, 236]. In addition, both HBsAg and HBcAg can achieve antiviral immune escape by activating the TLR2 signaling pathway in macrophages. The difference is that HBsAg inhibits IL-12 production by macrophages for immune escape, whereas HBcAg inhibits antigen-specific CD8 + T cells by promoting IL-10 production by macrophages [237, 238].

##### NK cells

In chronic HBV and HCV infections, downregulation of activating receptors (e.g., NKG2D, NKp30, NKp46, etc.) and upregulation of inhibitory receptors (e.g., NKG2A, KIR, PD-1, Tim-3, etc.) in NK cells was observed, resulting in impaired function of NK cells. This is manifested by impaired secretion of cytokines such as IFN-γ without a significant decrease in cytotoxicity [239, 240]. This phenomenon is known as the “functional dichotomy” of NK cells [241, 242]. In addition, chronic HCV infection induces the shedding of CD16 receptors mediating ADCC on NK cells, which may impair the ADCC function of NK cells and promote immune escape of HCV virus [243]. In addition to the effects of altered receptor phenotype on NK cells, various immune cells such as Treg cells and Breg cells [239, 244, 245], also keep NK cells in a state of exhaustion, mainly in the form of a marked decrease in IFN-γ production capacity [246]. During chronic HBV infection, activated NK cells are able to delete specific T cells, leading to persistent viral infection [241, 244]. Similarly, in chronic HCV infection, the enhanced effect of cytotoxicity of NK cells on T cells induced by CD14 + monocytes-derived hemagglutinin-9 may be associated with liver injury and persistent infection in chronic HCV infection [247]. Recent evidence suggests that there is an immunosuppressive cascade that mediates the expression of high levels of PD-1 by NK cells and the secretion of IL-10 to achieve suppression of T cells in chronic HBV infection; Suppressive monocytes induced by hepatitis B surface antigen (HBsAg) confer such properties to NK cells [248].

##### Adaptive cellular immune

The role of the adaptive immune response in the clearance of viruses or control of viral infections is widely recognized. During chronic HBV and HCV infection, the adaptive immune response is extensively disrupted by multiple mechanisms and this phenomenon may persist after reduction or elimination of the virus by direct antiviral therapy [249]. There are two main mechanisms that may contribute to the failure of virus-specific T cells response: T cells exhaustion and viral escape mutations [250].

One mechanism of low antiviral responses of T cells is the depletion of virus-specific CD8 + T cells due to persistent HBV and HCV infection, characterized by high expression of suppressive immune checkpoints, including PD-1, CTLA-4, lymphocyte activation gene 3 (LAG-3), T cell membrane 3 (Tim-3) and CD244 (2B4) [251, 252]; as well as a reduction in direct and indirect cytotoxic effects and a decrease in cytokine release [253]. Blocking the PD-1/CTLA-4 pathway was able to partially reverse the immune effects of HBV-specific CD8 + T cells depletion [254], however, recent studies point out that its clinical therapeutic effects may be limited [255]. Treg cells up-regulate PD-1 on T cell surface and impair its secretion through effector molecules such as IL-10 [256, 257]. MDSCs are another type of immunosuppressive cells that suppress T cells responses and are associated with viral persistence in patients with chronic HBV infection [258, 259].

During CVH, abnormalities in CD4 + T cells are mainly characterized by reduced cell numbers, high expression of immune checkpoint receptors (e.g., PD-1, CTLA-4, and LAG-3) [260, 261], reduced cytokine secretion (e.g., IFN-γ, IL-2, and TNF-α), and poorly differentiated T cells subpopulations [262, 263]. Tfh cells are an effector subpopulation of CD4 + T cells that promote the differentiation of B cells into antibody-secreting plasma cells [264, 265]. In mouse models and in patients with persistent HBV infection, Treg cells have been shown to inhibit Tfh cells-mediated HBV clearance [266]. In a recent clinical trial, the impaired function of Tfh cells was effectively improved with TLR8 agonists, thereby restoring HBV-specific B-cells responses [267].

Studies of defective B cells are limited. Although HBsAg-specific B cells have been identified in the blood and liver of many patients with chronic HBV infection, they exhibit defects in antibody secretion [268, 269]. The accumulation of atypical memory B cells (atMBC) may explain this phenomenon, as the high PD-1 expression on the surface of these cells may impair B cells immunity [268]. In addition, in chronic HBV infection, Breg cells may mediate T-cells immune abnormalities through IL-35, the number of which positively correlates with serum ALT and HBV viral load [270, 271]. The HCV-specific antibodies primarily target E1 and E2 envelope proteins, has viral neutralizing activity but is also susceptible to loss of neutralizing activity due to viral immune escape [272, 273].

CVH patients have antiviral immune deficiency involving IFNs, innate and adaptive immune system abnormalities. The mechanisms by which the virus evades immune surveillance are becoming clear, but the mechanisms by which the virus affects the immune system are still worth investigating, which is directly related to the combination of direct antiviral drugs with immune boosters and the restoration of antiviral immunity after antiviral therapy. In addition, the restoration of antiviral immunity still needs to consider the problem of liver inflammation and deterioration of liver function in CVH, and research in this area requires the accumulation of data from clinical studies and further basic research.

#### Hepatocellular carcinoma: immunosuppressive tumor microenvironment

HCC is a significant cause of cancer-related deaths, causing nearly 800,000 deaths worldwide in 2018 [274]. As the majority (80–90%) of HCC cases occur in a chronic hepatic inflammatory setting (e.g., chronic hepatitis B and C, alcoholic and non-alcoholic liver disease, liver fibrosis/cirrhosis) [275–279], it is considered to be the prototype of inflammatory cancers caused by chronic liver injury.

Although the respective microenvironments of alcoholic and nonalcoholic liver disease, chronic viral infection and HCC have been intensively studied, little is known about the transition from the microenvironment of chronic liver diseases to the tumor immune microenvironment (TME). Among them, the precancerous inflammatory factors of chronic liver diseases (chronic liver injury, inflammation and fibrosis) driving the development of TME has been a matter of interest [280]. Unlike precancerous inflammatory factors, recent studies suggest that pathogenic factors of chronic liver disease (i.e., precancerous non-inflammatory factors) such as (viral infection, alcohol, lipid metabolism, gut microbes) can directly affect immune cells in TME without undergoing inflammation-related processes [280, 281] (Fig. 4c). Tumor tissues and cells can also further shape the immune landscape of TME through complex interactions with immune cells [282]. In addition, adaptive immune cells that undertake immune surveillance functions, such as NK cells [283], CD8 + T cells [199], Th17 cells [284, 285] and B cells [286] have their own deleterious aspects of promoting tumor development, which are discussed in detail in some excellent reviews [287–289].

#### Factors influencing the formation of TME

##### Tumor cells and tissues

HCC cells can mediate TME immunosuppression and autoimmune escape through various mechanisms leading to dysfunction of effector cells such as T cells and NK cells [282]. For instance, full T cells activation requires co-stimulation of B7 molecules on APCs and CD28 molecule receptors on T cells, whereas HCC downregulates the expression of co-stimulatory molecule receptors such as B7.1 / B7.2, leading to tumor immune escape [290]. Yang et al. demonstrated that tumor cells-derived Wnt ligands stimulate the polarization of M2-TAMs through classical Wnt/β- catenin signaling, which leads to immunosuppression in HCC [291]. Furthermore, culture supernatants of the hepatoma cell line Huh7 appear to promote CD4 + CD25 + Treg cells proliferation and inhibit CD4 + CD25- T cells proliferation [292].

Tumor-derived exosomes (TEXs) mediate communication and interactions between tumor cells and immune cells and are an important way for tumor cells to promote TME formation [293–295]. In HCC, HCC-derived exosomes lead to impaired antitumor capacity of tumor-infiltrating T lymphocytes (TILs), which may be related to the delivery of 14–3-3ζ protein from HCC cells to T cells via exosomes [296]. In addition, Ye et al. found that the high mobility group box 1 (HMGB1) protein in EVs promoted T cells immunoglobulin and mucin domain 1 (TIM-1) regulatory B cells expansion and suppressed CD8 + T cells proliferation as well [297].

For quite some time, cell–cell fusion between immune cells and tumor cells has been hypothesized to be a mechanism promoting tumorigenesis and especially metastasis [298, 299]. A recent classical study confirmed by multiple discrete evidences the production of hybrid fusions by fusion of macrophages with tumor cells and found that such cells fusion products do exist in tissue specimens from different patients with solid tumors and in circulation [300]. This hybrid fusion confers enhanced tumorigenic potential and growth advantage to the tumor cells, tissue metastasis and immune privileges due to the macrophage identity [300]. Moreover, physical conditions within the tumor such as ECM stiffness, low pH, and additional factors such as hypoxia and high interstitial fluid pressure also tend to suppress the recruitment and function of anti-tumoral immune cells [301, 302].

##### Precancerous inflammatory factors

The pre-cancerous environment (PME) characterized by inflammation at the core of chronic liver diseases consists mainly of chronic liver injury-mediated hepatic oxidative stress, inflammation, fibrosis, and DNA damage in hepatocytes [280]. On the one hand, the sustained expression of cytokines (e.g. IL-1, IL-6, TNF-α and lymphotoxin β) [303] and the recruitment of immune cells in the context of a chronic inflammatory state may lead to DNA damage and in some cases to epigenetic alterations leading to mutations and tumor transformation [304–306]. Importantly, chronic inflammation leads to the production of growth factors that promote the growth of new tumors, making them appear as “non-healing wounds”[281]. On the other hand, chronic inflammation induces changes in the phenotype and effects of immune cells that exert anti-tumor activity, allowing cancer cells to evade hepatic immune surveillance [281]. For example, pro-inflammatory signals such as the chemokine axis CCR6-CCL20, IL-10 and TGF-β promote the activation of immunosuppressive Treg cells [307, 308].

Another prominent features of HCC is its strong association with liver fibrosis, with 80–90% of HCC occurring in fibrotic or cirrhotic livers [309]. HCC is closely associated with liver fibrosis and cirrhosis, suggesting that the pre-cancerous fibrotic environment of HCC may influence tumor formation [310]. In chronic HBV infection, HBV-specific CD8 + T cells have been identified as key players in the antiviral response, and extremely activated CD8 + T cells induce a huge inflammatory response and subsequent fibrosis that can promote hepatocarcinogenesis [311, 312]. Recently, it has been shown that HSCs increase the levels of Th17 cells and upregulate Treg cells, which may contribute to the development of HCC after HBV cirrhosis [313]. Hence, chemopreventive strategies that reduce inflammation and inhibit the initiation or propagation of ongoing inflammation may prevent or delay cancer development [304, 314]. For example, the regular clinical use of non-steroidal anti-inflammatory drugs (NSAIDs) that inhibit inflammation, such as aspirin, has been associated with a reduction in the incidence of hepatocellular carcinoma [315–318]. In addition, aspirin may be used as an adjuvant to other therapies to reduce recurrence of hepatocellular carcinoma [319].

##### Precancerous non-inflammatory factors

As mentioned above, the stage of chronic liver disease into cirrhosis is closely related to the development of HCC, and it is estimated that 80% of HCC occurs in the context of cirrhosis [309]. However, 20% still occur in the context of non-cirrhotic liver diseases, called non-cirrhotic hepatocellular carcinoma (NCHCC) [320]. This laterally suggests that non-inflammatory factors prior to HCC formation are directly involved in HCC development and lead to HCC-specific TME formation.

***Chronic viral infection.*** Viral infections are associated with NCHCC. Individuals with NCHCC have hepatitis B core antibodies and occult HBV infection, suggesting a role for hepatitis B infection in NCHCC [321]. In Asia, a data from Korea showed that the main cause of NCHCC was HBV infection (77.2%) [322]. HBV-infected hepatocytes do not trigger significant fibrosis or inflammation in the liver, which is a characteristic manifestation of HBV-induced NCHCC [323]. HBx proteins can initiate epigenetic modifications to dysregulate miRNAs expression, which in turn can regulate downstream epigenetic changes in HBV-HCC pathogenesis, demonstrating the complex interplay between HBV infection, epigenetic changes, disease and immune response [324].

***NAFLD/NASH.*** Patients with NAFLD, especially those with fibrosis or progressing to cirrhosis, are at increased risk of progression to NAFLD-related HCC, but 20–50% of NAFLD-related HCC cases still occur in the absence of advanced fibrosis [325, 326]. Multiple analyses of data from clinical settings suggest that NAFLD is a major cause of NCHCC [323, 327]. There is research to prove that dysregulation of lipid metabolism in NAFLD induces hepatic accumulation of linoleic acid and subsequent loss of CD4 + T cells due to increased reactive oxygen species (ROS), leading to an increased incidence of HCC [328]. In addition, obesity, a risk factor for NAFLD, impairs the function of CD8 + T cells and enhances the immunosuppressive potency of tumor-infiltrating MDSCs [329, 330]. Metabolic therapy may play a role in the prevention of HCC in patients with hepatic steatosis and concomitant liver diseases [331]. Recent evidence suggests that bacterial extracts from the NAFLD-HCC microbiota, trigger a T cells immunosuppressive phenotype characterized by the expansion of IL-10 secreting Treg cells and attenuation of CD8 + T cells, at least in part through increased production of short-chain fatty acids [332]. Both obesity and gut microbes contribute to the accumulation of hepatic bile acids (BA) and metabolites [333, 334], and inhibition of 7α-dehydroxylation, which is responsible for secondary BA metabolism, is associated with a low incidence of HCC in mice [335].

##### TME in HCC

The microenvironment of HCC is characterized by an immunosuppressive environment of immune cells and tumor vasculature that is structurally and functionally abnormal [336]. Briefly, the immune cells and cellular mediators are profoundly altered in the immunosuppressive microenvironment of HCC, especially tumor-specific immunosuppressive cells, including TAMs, TANs, CAFs, MDSCs and Treg cells, which promote tumor development as well as metastasis [337]. The upregulation of immune checkpoints, e.g., PD-1/PD-L1 and CTLA-4, is one of the mechanisms by which cancer suppresses antitumor immune responses, and these cells granzyme B and effector cytokine levels are reduced [251].

In conclusion, studies on the factors affecting anti-tumor immune cells during the progression of chronic liver diseases to HCC and the molecular mechanisms behind this shift in the pro-tumor effect of immune cells are inadequate, and we briefly describe the pre-cancerous inflammatory and non-inflammatory factors and the role of tumor tissue and cells in TME formation, information that is essential for the prevention of HCC development by boosting anti-tumor immunity and for the treatment of HCC.

## Current status of immunotherapy for liver diseases

As mentioned above, abnormalities in the hepatic immune cell microenvironment are directly involved in the development of liver diseases, and therapeutic strategies targeting these immunopathogenic pathways are increasingly being used in preclinical and clinical studies, showing good promise. We summarized representative therapies with clinical potential for various liver diseases and their mechanisms (Table 1). Although some immunotherapies showed some potential in preclinical studies, the phenotypes were not satisfactory in clinical studies. For example, therapeutic agents targeting inflammation and fibrosis in NASH, CCR2/CCR5, TLR4, ASK1 and lysine oxidase were considered ineffective in recent clinical trials to alleviate endpoint outcomes in NASH, particularly fibrosis [10, 338]. Furthermore, although several smaller clinical trials in patients with end-stage liver disease of varying severity have shown that G-CSF improves patient survival and reduces complications [339]. However, in a recent multicenter controlled trial, G-CSF did not have a significant beneficial effect in patients with chronic acute liver failure, suggesting that it should not be used as standard of care for end-stage liver diseases [340].

**Table 1:** Current representative and promising therapeutic strategies targeting immunopathogenesis in liver diseases

Diseases	Immunotherapy	Specific components	Treatment mechanism	Reference
**AIH**	Corticosteroids	Predniso(lo)ne and Budesonide	Affect T cells gene transcription upon binding to the glucocorticoid receptor	[341]
	Inhibitors of purine synthesis	Azathioprine and Mycophenolate Mofetil	Interfere with the S-phase of the cell cycle, leading to T cells death	[341]
	Calcineurin inhibitors	Ciclosporin and Tacrolimus	Act on the calcineurin calmodulin complex to inhibit IL-2 gene expression and Teff activation	[341]
	Mammalian target of rapamycin (mTOR) inhibitors	Rapamycin and its analogues	Act on the mTOR inhibits cell cycle progression, thereby inhibiting effector T and B cells proliferation	[341, 342]
	Targeting B lymphocytes	Anti-CD20 monoclonal antibodies	B cells depletion and reduced autoantibody production	[343, 344]
		Anti-BAFF receptors	Reduce the proliferation and differentiation of B cells by soluble BAFF	[343]
	Targeting inflammatory cytokines	Anti-TNF-α	Directly neutralizes soluble TNF-α and has pro-apoptotic and anti-proliferative effects on lymphocytes	[341, 345]
	Adoptive transfer of cells	Adoptive transfer of Treg cells	Teff cells effect was inhibited by secretion of IL-10, TGF-β, IL-35 and direct cytotoxicity	[341, 346]
	Autoantigen specific immunotherapy (ASIT)	Autoantigen particle presentation system	Target tolerogenic DCs or direct antigen to hepatic APCs, inducing antigen-specific T cells incompetence	[347, 348]
**AH**	Corticosteroids	Prednisone	Non-specific reduction of pro-inflammatory cytokines and increase of anti-inflammatory cytokines	[349]
	Targeting inflammatory cytokines	IL-1 receptor antagonist (Anakinra); IL-1β antibody (canakinumab)	Inhibit IL-1 signal transduction, inhibit the activation of liver macrophages, and reduce liver inflammation	[350–352]
	Targeting immune cells recruitment	Spleen tyrosine kinase (SYK) inhibitors	Reduces macrophage and neutrophil activation and recruitment, and reduces hepatic stellate cell activation	[353]
		Monocyte chemoattractant protein-1 (MCP-1) inhibitors	Reduce macrophage recruitment and reduces liver inflammation by inhibiting MCP-1	[354, 355]
		Chemokine CCR2/5 antagonists (cenicriviroc)	Prevent the increase in infiltrating Macrophages (F4/80^low^CD11b^hi^) and reduced proinflammatory Ly6C^hi^ Macrophages in livers	[356]
	Targeting TLRs signaling pathways	TLR4 antagonists	Inhibits activation of TLR4 signaling pathway in inflammatory cells such as macrophages and reduces liver inflammation	[357]
		Probiotics (e. g. Lactobacillus rhamnosus GG)	Reduce the expression of TLRs and intestinal microbe stimulation to TLRs, reduce inflammatory factors	[356, 358]
	Targeting PD-1 + and Tim-3 + T cells	Immune checkpoint inhibitors (ICIs)	Restore T cells production of interferon gamma, reduce the numbers of IL-10 producing T cells, and increase neutrophil antimicrobial activities	[359]
**NASH**	Farnesoid X receptor (FXR) agonists	Obeticholic acid (OCA)	Inhibit NLRP3 inflammatory vesicle activation in macrophages and further inhibits hepatic lipid accumulation triggered by inflammatory vesicle activation	[360]
	Targeting vascular cells adhesion molecule-1 (VCAM-1)	VCAM-1 inhibitor	Reduction of mononuclear macrophage infiltration by inhibiting monocyte adhesion to LSECs	[361, 362]
	Clearing senescent cells	Urokinase-type plasminogen activated receptors (uPAR) -specific CAR-T cells	Specifically eliminates senescent HSCs and macrophages, inhibits inflammation and fibrosis caused by senescent cells	[363, 364]
	Dual TBK1/IKKε inhibitors	amlexanox	Inhibit activation of Kupffer cells and induces polarization of Kupffer cells to the M2 phenotype; inhibit activation of HSCs	[365–367]
**Cirrhosis**	Specific inhibitor of TLR4 receptors	TAK-242	Inhibition of LPS-mediated TLR4 signaling, which in turn inhibits inflammatory cells, HSCs activation and liver injury	[368]
	Specialized proresolving mediators (SPM)	Lipoxins, Resolvins, Protectins and Maresins	Inhibits neutrophil recruitment, promotes macrophage polarization to the M2 phenotype and secretion of MMP-9, and promotes macrophage autophagy	[369, 370]
	Targeting macrophages	Autologous macrophage infusion	May be related to inhibition of inflammation and hepatic stellate cell activation and promotion of ECM degradation	[216, 371]
		Notch antagonists (Avagacestat); DPP4 antagonists (anagliptin);	Polarising anti-inflammatory M2 macrophages	[372, 373]
	TLR7/8 agonists	R848	Restore and maintain the function of neutrophils against infection, induce the polarization of liver M2 macrophages, and play the role of liver repair and anti-fibrosis	[374, 375]
**CVH**	Peg-interferon	Pegylated interferon alpha	Induce innate and adaptive antiviral immune effects in the body and inhibits viral replication	[376–378]
	Pattern recognition receptors (PRR) agonists	TLR7–8 ligands	Promote IFN-α production in hepatocytes and immune cells, induce interferon-related genes (ISGs) expression; regulates virus-specific T cell and B cell responses; reduce hepatitis B virus cccDNA load	[379–381]
	Immune checkpoint inhibitors (ICIs)	PD-1/ PD-L1 monoclonal antibodies	Restoration of virus-specific T cells function and acquisition of sustained immunological control of viral infections	[382, 383]
		Cytotoxic T Lymphocytes-Associated Antigen-4 (CTLA-4) monoclonal antibodies	Restoration of virus-specific T cells function and acquisition of sustained immunological control of viral infections	[254, 384]
	Therapeutic vaccines	Viral antigens and protein vaccines, vaccines based on viral vectors, anti-HBsAg antibodies, DNA vaccination	Vaccination with non-infectious forms of viral antigens as a means of inducing or enhancing existing virus-specific immune responses	[385]
	Adoptive transfer of cells	Autologous T cells, CAR-T cells	Infusion of virus-specific T cells enhances the body's antiviral immunity	[386, 387]
**HCC**	Immune checkpoint inhibitors (ICIs)	PD-1/PD-L1 and CTLA-4 monoclonal antibodies	Block the inhibitory signals between tumor cells and immune cells by infusing monoclonal antibodies to the corresponding immune checkpoints, thereby improving anti-tumor efficacy	[388]
	Adoptive cellular therapy (ACT)	Cytokine-induced Killer Cells (CIK)	Produced by in vitro expansion of peripheral blood mononuclear cells (PBMC), consisting mainly of NKT cells, NK cells and CTLs; recognize tumor cells by adhesion molecules and lyse them in a major histocompatibility complex (MHC)-independent manner	[389]
		Tumor-Infiltrating Lymphocytes (TILs)	Composed of a mixture of cell types isolated from tumor specimens, foxp3 + , CD8 + , CD3 + , and CD4 + T lymphocytes are the broadly studied subgroups of TILs, whose TCRs recognize multiple tumor antigens	[390]
		Chimeric antigen receptor-T cells (CAR-T); Chimeric antigen receptor -NK cells (CAR-NK)	Genetic modification of T cells or NK cells to express receptors that recognize specific antigens, activate upon binding to external antigens, and induce perforin- and granzyme-mediated apoptosis	[391]
		TCR-T cells	T cells with high affinity for tumor-specific antigens are screened and isolated, and the desired TCR gene sequence is inserted into T cells, which are isolated and amplified and then reinfused to perform anti-tumor functions	[392]
	Therapeutic vaccines	Nucleic acid, antigenic peptide, DCs and lysozyme vaccines	Vaccination in cancer therapy happens through in vivo delivery of specific TAAs, tumor lysates, and nontumor-associated individual antigens. These vaccine antigens are then processed by APCs such as DCs, and then coupled to MHC class I and II molecules to activate CTLs and CD4 + helper lymphocytes	[393]

## MSCs-based immunotherapy for liver diseases

**Fig. 5:**
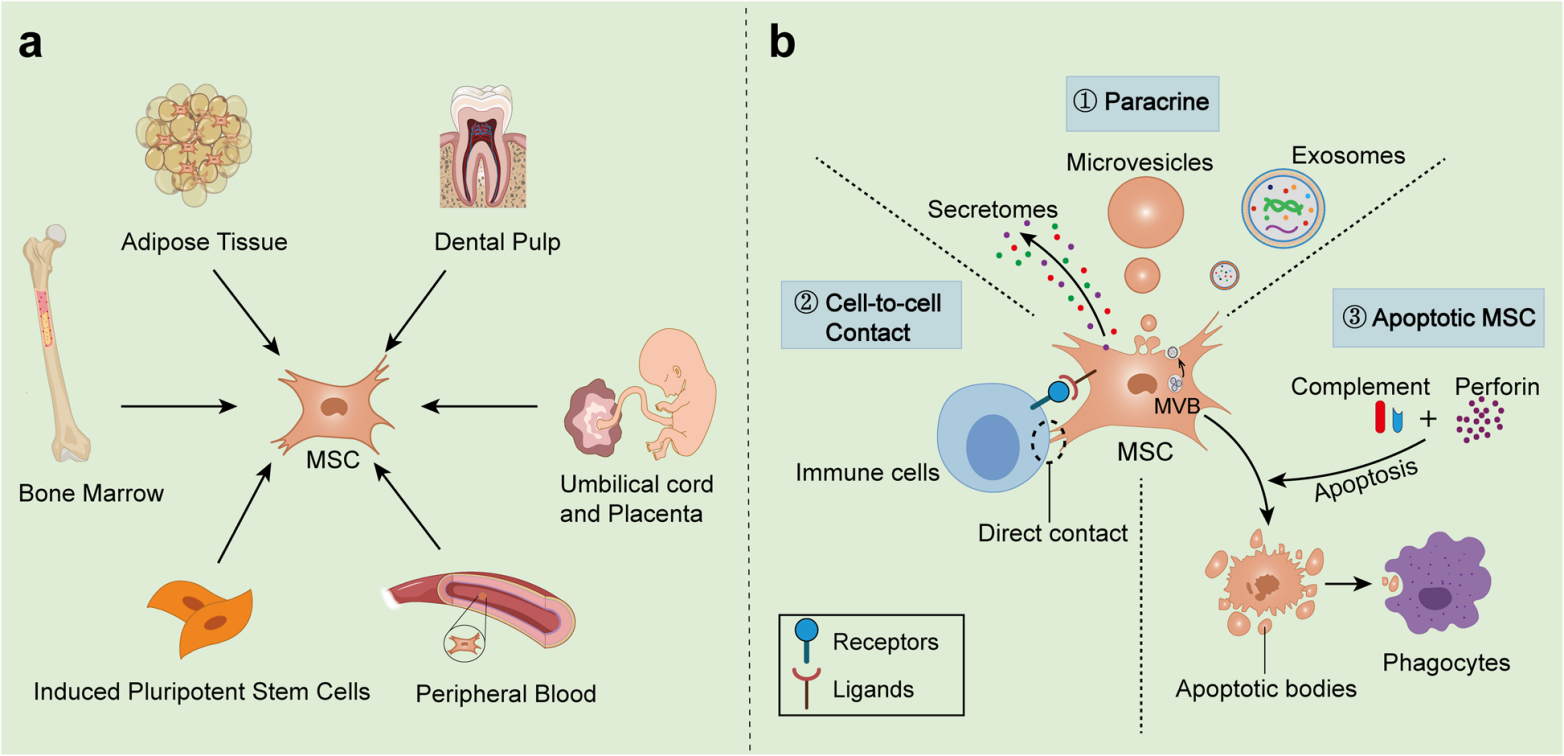
Sources of MSCs and the Pathways of MSCs exerting immunomodulatory effects. **a** MSCs can be isolated from the bone marrow, adipose tissue, dental pulp, umbilical cord and placenta, peripheral blood, and induced pluripotent stem cells. **b** There are three main pathways for MSCs to exert immunomodulatory effects: ① Paracrine effects, including microvesicles, exosomes and bioactive molecules; ② Cell-to-cell contact, including direct intercellular contact and intercellular contact mediated by the binding of cell surface receptors and ligands; ③ Apoptotic MSCs: MSCs are attacked by complement system components, complement activated neutrophils and perforin positive cytotoxic cells, then phagocytes engulf apoptotic MSCs to mediate immune regulation

MSCs are stem cells with multidirectional differentiation potential that can be isolated from the bone marrow, adipose tissue, dental pulp, umbilical cord and placenta, peripheral blood, and induced pluripotent stem cells [394, 395] (Fig. 5a), easy to obtain and culture, and have low immunogenicity; moreover, MSCs-derived exosomes are increasingly used for cell-free therapy and therapeutic vectors. Based on these advantages MSCs are increasingly used for the treatment of liver-related diseases [12]. The mechanism of MSCs in the treatment of liver diseases is mainly attributed to their immunomodulatory and liver tissue regenerative abilities, in addition to their direct anti-fibrotic effects and good homing properties to inflammatory injury and tumor sites, making them the star cells for the treatment of liver diseases [396].

Currently, there are 62 ongoing clinical trials using MSCs for different liver diseases [Accessed January 01, 2022]https://www.clinicaltrials.gov/. The studies involved a variety of liver diseases, including AIH (*n* = 2), acute liver failure (*n* = 17) and cirrhosis (*n* = 43) caused by alcohol, hepatitis B or C virus infection, NASH and primary biliary cholangitis, as well as unspecified causes. There was significant heterogeneity in these clinical studies in terms of dose and dose of injected cells, source of stem cells, type of transplantation, route of injection, and study design. We summarize in detail 20 of these clinical trials with clear etiology, including AIH (*n* = 2); Liver cirrhosis caused by alcohol (*n* = 2), hepatitis B (*n* = 3), hepatitis C (*n* = 1), hepatitis C or NASH (*n* = 1), Primary biliary cirrhosis (PBC) (*n* = 4); and Liver failure caused by hepatitis B (*n* = 6), alcohol (*n* = 1) (Table 2). They have demonstrated safety and efficacy in clinical treatment.

**Table 2:** Summary of clinical trials on mesenchymal stem cells for liver diseases

Liver Disease and cause	Start time (year)	Type of clinical trials	Source of MSCs	Route	Dose of MSCs	Phase	ClinicalTrials.gov identifier
Autoimmune hepatitis	2011	Parallel-controlled trial	Allogenic, umbilical cord	Peripheral vein	1 × 10^6^ cells / kg body, 4 doses	1/2	NCT01661842
	2018	Parallel-controlled trial	Allogenic, umbilical cord	Peripheral vein	combination of 0.5, 1.0, 2.5 million cells / kg body (3 dose levels), single infusion	2a	NCT02997878
Liver cirrhosis caused by alcohol	2019	No control group	Autologous, bone marrow	Hepatic artery	5 × 10^7^ cells / 10 mL, single infusion	1	NCT03838250
	2021	Randomized controlled trial	Autologous, bone marrow	Hepatic artery	7 × 10^7^ / dose, single infusion	3	NCT04689152
Liver cirrhosis caused by hepatitis B	2012	Parallel-controlled trial	Allogenic, umbilical cord	Hepatic artery	1 × 10^6^ cells / kg body, single infusion	1/2	NCT01728727
	2018	No control group	Autologous, bone marrow	Peripheral vein	0.5–1 × 10^6^ cells / kg body, single infusion	3	NCT05080465
	2018	No control group	Autologous, umbilical cord	Peripheral vein	100 million cells / dose, single infusion	1/2	NCT04357600
Liver cirrhosis caused by hepatitis C	2016	No control group	Autologous, adipose	Peripheral vein or Hepatic artery	1 million cells / kg body, 3 doses in the Peripheral vein and 3 million cells / kg body, 3 dose into the right hepatic artery	1/2	NCT02705742
Liver cirrhosis caused by hepatitis C or non-alcoholic steatohepatitis (NASH)	2017	No control group	Autologous, adipose	Peripheral vein	None	1/2	NCT03254758
Primary biliary cirrhosis (PBC)	2011	Parallel-controlled trial	Autologous, umbilical cord	Peripheral vein	1 × 10^6^ cells / kg body, 3 doses	1/2	NCT01662973
	2011	Parallel-controlled trial	Autologous, bone marrow	Peripheral vein	5–50 million cells / kg body, single infusion	1	NCT01440309
	2017	Parallel-controlled trial	None	Peripheral vein	0.1–1 × 10^6^ cells / kg body, 3 doses	None	NCT03668145
	2019	Paired, controlled study	Autologous, umbilical cord	Hepatic artery	1 × 10^6^ cells / kg body, 2 doses	1/2	NCT04522869
Liver failure caused by hepatitis B	2013	Parallel-controlled trial	Autologous, umbilical cord and bone marrow (2 experimental groups)	Peripheral vein	combination of 1 × 10^5^ cells / kg body, 1 × 10^6^ cells / kg body, 1 × 10^7^ cells / kg body (3 dose levels), 8 doses	1/2	NCT01844063
	2016	Parallel-controlled trial	Autologous, umbilical cord	Peripheral vein	None	2	NCT02812121
	2017	Randomized controlled trial	Autologous, None	Peripheral vein	1 × 10^6^ cells / kg body, 3 doses	None	NCT03209986
	2019	Parallel-controlled trial	Autologous, umbilical cord	Peripheral vein	1 × 10^6^ cells / kg body, 3 doses	2	NCT03945487
	2019	Parallel-controlled trial	Autologous, human exfoliated deciduous teeth	Peripheral vein	1 × 10^6^ cells / kg body, 4 doses	1	NCT03957655
	2021	No control group	Autologous, umbilical cord	Peripheral vein	1 × 10^8^ cells / dose, 2 doses	None	NCT05106972
Liver failure caused by alcohol	2009	No control group	Autologous, bone marrow	Hepatic artery	5 × 10^6^ cells / mL, 2 doses	2	NCT01741090

### Immunomodulatory capacity of MSCs

The immunomodulatory capacity of MSCs is not immutable, but is modified according to the different and evolving microenvironments to which they are exposed, and is referred to as immune plasticity [14, 397]. MSCs can promote inflammation when the immune system is under-activated and suppress inflammation when the immune system is over-activated in order to avoid self-attack. This activity is also referred to as a function of “sensors and switches of the immune system” [15]. Different levels of inflammatory factors (including IFN-γ, TNF-α and IL-1β, etc.) and specific types of activated TLRs determine the immunophenotype of MSCs [398–401]. Specifically, TLR4-activated MSCs exhibit a pro-inflammatory/tumor suppressive MSC1 phenotype, whereas TLR3-activated MSCs exhibit an immunosuppressive MSC2 phenotype [402, 403]. In conclusion, the ability of MSCs to exert opposing compensatory immunomodulatory effects in microenvironments with different levels of inflammation underlies their use in the treatment of liver diseases mediated by immune imbalance.

The plasticity of MSCs immunomodulation has enabled MSCs to show an irreplaceable role in the immunotherapy of acute and chronic liver-related diseases. Numerous preclinical and clinical studies have demonstrated that MSC-based immunosuppressive therapies can effectively suppress immune hyperactivation in inflammatory liver diseases, thereby reducing immune-mediated liver injury, inflammation and fibrosis [404, 405]. In addition, in viral infections and oncological diseases, the application of MSCs is still relatively rare and clinical studies are lagging behind due to concerns about the risk of virus carriage and tumorigenesis. However, in recent years, indirect immunotherapeutic strategies based on MSCs platforms, especially the adaptation of MSCs and their extracellular vesicles into targeted delivery systems for therapeutic drugs or immunomodulators by genetic modification and other physical or chemical means, can enhance antiviral and antitumor immunity in CVH and HCC and effectively circumvent their deleterious properties [22, 406, 407].

### Pathways of immunomodulatory function of MSCs

The powerful immunomodulatory capacity of MSCs has been demonstrated and is a major advantage for their use in the treatment of liver diseases, especially immune inflammatory liver diseases. It has immunomodulatory capacity on a wide range of innate and adaptive immune cells in the liver and in the circulation [408, 409]. The immunomodulatory function of MSCs is mainly exerted through interactions with immune cells, including direct or indirect intercellular contacts, and paracrine effects [410–412] (Fig. 5b). MSCs modulate the host immune system through paracrine signaling [14, 411], including the secretion of soluble molecules, or release of more complex structures called extracellular vesicles (EVs) [413]. Specifically, the MSCs can secrete myriad growth factors [414], chemokines [415], and immunomodulatory cytokines ( such as IL-10 [416], IDO [417, 418], HO-1 [419], TSG6, PGE2 [420, 421]), and other soluble molecules. Moreover, EVs are cell membrane encapsulated vesicles, including exosomes, microvesicles and apoptotic vesicles, which can carry various bioactive molecules, including lipids, proteins, DNA and RNAs such as microRNAs. These contents are transported and released into immune cells to regulate their phenotype and function [422–424].

In addition, recent findings suggest that apoptotic, metabolically inactivated, and even fragmented MSCs also show immunomodulatory potential [425, 426] (Fig. 5b). Back in 2005 Thum et al. published the “dying stem cell hypothesis”, which suggested that apoptosis of bone marrow MSCs causes modulation of local immune responses, leading to downregulation of innate and adaptive immunity [427]. In later studies, apoptotic adipose tissue-derived MSCs (A-ADMSCs) were shown to be effective in treating organ injury in sepsis models, reducing inflammation, fibrosis and apoptosis levels [428, 429]. Importantly, compared to surviving MSCs implanted in different tissue microenvironments, apoptotic MSCs showed no change in immunomodulatory properties across stimuli, suggesting their potential for clinical application [430].

Traditionally, transplanted MSCs were attacked by components of the complement system, complement-activated neutrophils and perforin-positive cytotoxic cells, inducing them to undergo apoptosis. Apoptotic MSCs can be taken up by phagocytes, producing immunosuppressive consequences [14]. A study using a mouse graft-versus-host disease (GvHD) model demonstrated that cytotoxic cells actively induce apoptosis in MSCs in a perforin-dependent manner. Further studies have shown that IDO, produced by phagocytes after phagocytosis of apoptotic MSCs, is essential for the initiation of immunosuppressive effects by apoptotic MSCs [431].

## MSCs for inflammatory liver diseases

### MSCs for autoimmune hepatitis

**Fig. 6:**
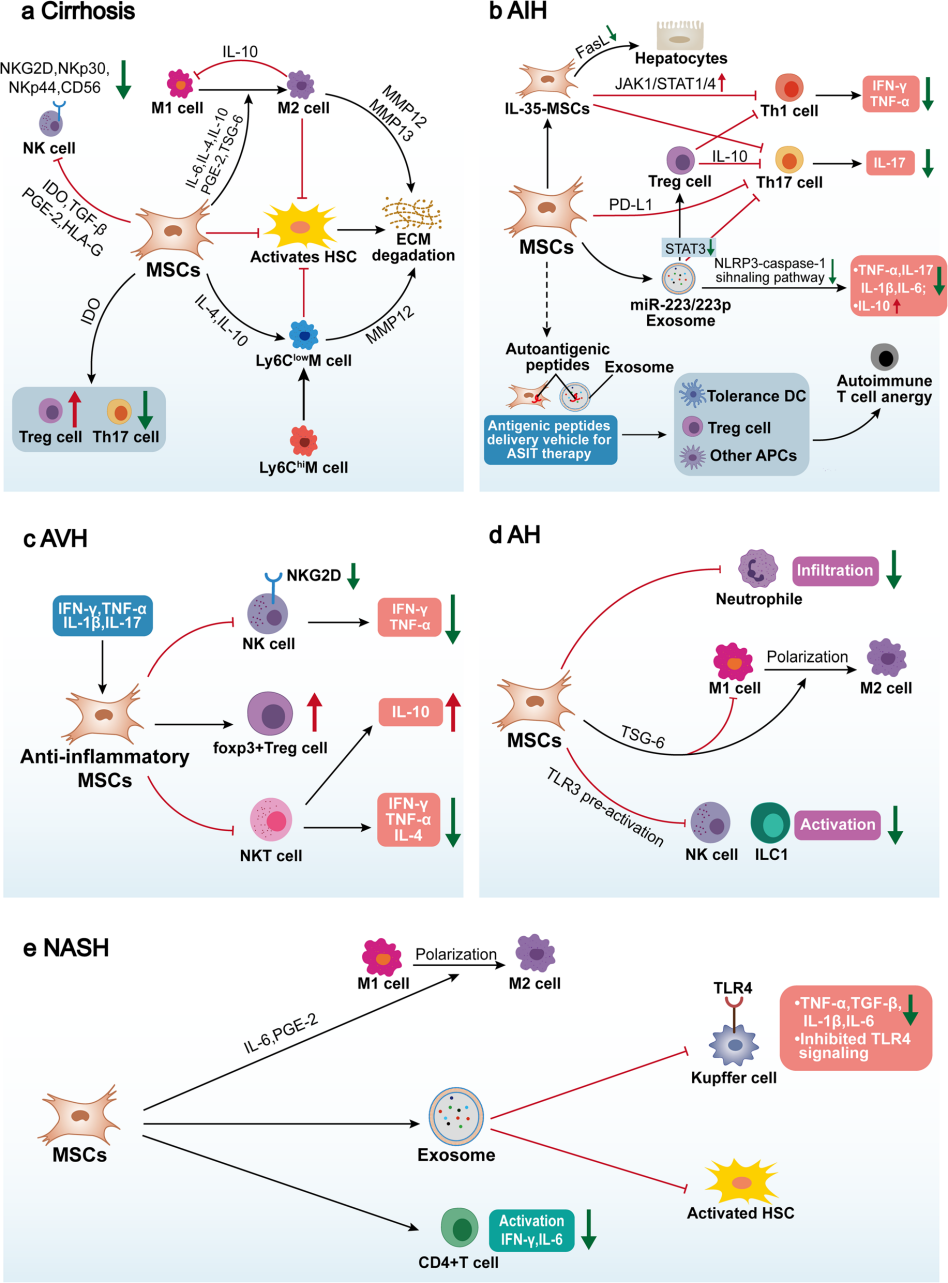
Immunomodulation and immunotherapeutic strategies of MSCs in liver diseases. Due to the immunomodulatory plasticity of MSCs and their ability to deliver immunomodulators after modification, MSCs can exert immunosuppressive effects in inflammatory liver diseases including (**a**) Cirrhosis, (**b**) AIH, (**c**) AVH, (**d**) AH and (**e**) NASH, thereby ameliorating immune-mediated liver injury, liver inflammation and fibrosis. The dashed arrows indicate potential immunomodulatory mechanisms or immunotherapeutic strategies

As previously mentioned, AIH is an autoimmune disease caused by the erroneous attack of T cells on their own hepatocytes and loss of immune tolerance. MSCs are promising candidates for the treatment of AIH with immunosuppressive properties that can reintroduce self-tolerance in the liver by correcting excessive immune responses [16, 432, 433]. Three MSCs clinical trials have been conducted (Table 2). Studies have shown that for autoimmune diseases, including AIH, the specific mechanisms by which MSCs exert their therapeutic effects lie in the inhibition of lymphocyte activation and proliferation and in the promotion of Treg cell formation [434, 435] (Fig. 6b). These abilities are associated with the expression/secretion of molecules such as CTLA-4, PD-L1, IDO-1, FasL, iNOS, TGF -β and PGE2 by MSCs [432].

Chen et al. earlier investigated the therapeutic effect of bone marrow MSCs (BM-MSCs) transplantation in experimental autoimmune hepatitis (EAH) in mice and showed that BMSCs transplantation, especially multi-dose transplantation, increased hepatic PD-L1 levels along with decreased IL-17 levels, producing immunosuppressive effects [436]. Similarly, a recent study used lentiviral transfection to construct PD-L1-high expressing MSCs-EVs, which successfully initiated immunosuppressive signaling in activated immune cells, including T cells, macrophages and DCs, by interacting with PD-1 on the surface of these cells [437]. By constructing mouse autoimmune disease models of ulcerative colitis and psoriasis, this study further revealed that extracellular vesicles with high PD-L1 expression could specifically target liver lesion sites and effectively modulate the balance between Th1, Th2, Th17 and Treg cells subsets to attenuate autoimmune responses in AIH [437].

IL-35 is known to belong to the IL-12 family, is produced by Treg cells and is required for the maximal suppressive activity of Treg cells [438]. Wang, et al. [439] showed hepatic by infusion of both IL-35 gene-modified mesenchymal stem cells (IL-35-MSCs) and adipose-derived MSCs in a mouse model of Con A-induced fulminant hepatitis protective effect. They found that MSCs could attenuate liver injury by reducing hepatic secretion of IL-17 but not IFN-γ, while IL-35-MSCs could not only reduce IL-17 secretion by Th17 cells but also reduce IFN-γ expression by Th1 cells through the JAK1-STAT1/STAT4 signaling pathway, as well as reduce hepatocyte FasL expression thereby preventing hepatocyte apoptosis [439]. In addition, MSCs-derived exosomes also have a therapeutic effect on AIH by regulating immunity of liver Treg and Th17 cells through specific miRNAs. It was shown that miR-223 in BM-MSCs-exos reversed liver inflammation and cell death by downregulating mRNA and protein levels of NLRP3 and caspase-1 in an experimental AIH model, while decreasing levels of pro-inflammatory cytokines (TNF-α, IL-17A and IL-1β) in serum and liver tissues [440]. The same group of investigators gave MSCs-exosomes overexpressing miRNA-223-3p had a significant attenuating effect on liver injury. BM-MSCs-exos increased the proportion of Treg cells but decreased the proportion of Th17 cells, while serum and liver levels of IL-1β, IL-6 and IL-17 were reduced, while IL-10 levels were increased. These results may be related to the downregulation of STAT3 by miR-223-3p [441].

The development of antigen-specific immune tolerance approaches for the treatment of autoimmune diseases has received much attention in recent years [347, 442, 443]. Antigen-specific immunotherapy (ASIT) aims to rebuild immune tolerance to autoantigens by providing the relevant antigen to a specific cell type or environment suitable for triggering a tolerogenic response while leaving the immune system to function effectively [444]. Liver antigen presenting cells, targeted by carrier particles, and steady-state dendritic cells, to which antigen-processing independent T cells epitopes(apitopes) bind selectively, as the principal targets for antigen-specific immunotherapy [348]. Apitopes induce tolerance through induction of anergy T cells and generation of Treg1 (Tr1) cells [445]. However, the core of ASIT design is the delivery of autoantigen peptides, and autoantigens and their constituent epitopes vary widely in physicochemical properties such as size, charge, and solubility, and these differences can greatly affect the systemic transport of ASIT agents [446]. MSCs, especially extracellular vesicles, are known for their low immunogenicity, biosafety and targeting properties have been used as in vivo delivery vehicles for therapeutic agents [447], and could theoretically be used as targeted delivery vehicles for ASIT agents to overcome possible limitations of in vivo factors such as susceptibility to digestion by enzymes in vivo, poor penetration across biological barriers, and rapid clearance by the reticuloendothelial system.

Regardless of preclinical and clinical trials, the therapeutic role of MSCs in AIH is receiving increasing attention and has demonstrated safety and efficacy. With further studies on the cellular and molecular mechanisms behind the changes in the immune microenvironment of AIH, the specific mechanisms behind the treatment of AIH with MSCs will be further validated. Importantly, based on the immunopathogenesis of AIH, the strategy of blocking immune damage in the liver by making MSCs transport immune-targeted drugs through genetic modification and physicochemical loading has good prospects for application.

### MSCs for acute viral hepatitis

Pharmacological interventions for AVH are extremely limited, including antiviral therapy. Currently known antiviral drugs are approved for chronic viral infections, while antiviral treatment regimens for acute viral infections remain undeveloped, especially in complex patients with acute liver failure and severe liver injury, for which there is still no consensus [448, 449]. Some studies point out that direct antiviral agents (DAAs) for acute HCV infection appear to have shorter treatment times and better outcomes [450, 451]. However, two meta-analyses assessing the efficacy of common pharmacological interventions for acute hepatitis B and C showed no significant benefit of antiviral drugs in preventing complications and disease progression compared to placebo, and other interventions, and varying degrees of adverse effects [452, 453]. In patients with acute severe hepatitis, especially acute fulminant liver failure, early liver transplantation remains the most effective clinical option [454]. Acute HBV or HCV infection’s can trigger severe liver damage and liver failure, which is associated with lysis of infected hepatocytes by the antiviral immune system. Therefore, further development of effective antiviral drugs to inhibit viral replication is needed, along with effective suppression of immune-mediated liver injury.

The initial use of MSCs in AVH has begun with the aim of controlling immune-mediated severe liver injury and maximizing the preservation of antiviral immunity (Fig. 6c). The treatment of viral infectious diseases with MSCs is predicated on their resistance to viral infection so as not to become a reservoir of viruses increasing the risk of virus transmission. It has been shown that MSCs are susceptible to viral invasion in vitro and in vivo [455, 456], but MSCs are well resistant to viruses, thanks in part to their regulation of the expression of their own ISGs [457]. Moreover, IDO is the main mediator that slows down viral replication in human MSCs, but the same effect was not observed in mice [458]. Therefore, the ability of MSCs to inhibit their own viral invasion and replication may depend on the different species origin.

It is well known that the immune regulation of MSCs is markedly plastic [397], and their immunosuppressive phenotype depends mainly on the stimulation of a certain combination of inflammatory factors, such as IFN-γ with TNFα or IL-1β [459]. In an acute HBV-infected mouse model, the relay transfer of bone marrow MSCs reduced NKG2D expression on NK cells, inhibited NK cells cytotoxicity in vitro and improved liver injury and liver inflammatory response. However, this resulted in enhanced hepatitis B virus gene expression and replication in vivo, although the impact of BM-MSCs on prolonged viral clearance needs to be considered in the future [460]. Treg cells are able to suppress effector T cells activity and innate cells recruitment during acute HBV infection, thereby attenuating immune-mediated liver injury [128]. Despite the lack of direct evidence in acute HBV or HCV infection, findings in a ConA-induced model of similar viral and immune-mediated liver injury suggest that MSCs exosomes significantly inhibit liver injury in model mice with an increase in Treg cells number [461]. In the same model, tonsil-derived MSCs (T-MSCs) can reduce liver injury by suppressing activated T cells through the secretion of Galectin-1 [462]. Besides, it was further shown that MSCs inhibit the secretion of TNF-α, IFN-γ and IL-4 from NKT cells in an NO- and IDO-dependent manner, but promote the secretion of their immunosuppressive cytokine IL-10, thereby reducing the additional liver damage caused by the immune system [463].

Therefore, in parallel with antiviral drug therapy, MSCs could theoretically be used in a timely manner for targeted treatment to reduce liver damage and liver failure in fulminant hepatitis. However, before the use of MSCs in AVH patients, reducing the risk of self-infection with MSCs and whether the immunosuppressive effect of MSCs impairs the body's ability to clear the virus, leading to persistent or chronic viral infection, as well as affecting the therapeutic efficacy of antiviral drugs, need to be further investigated. In patients with oncologic and autoimmune diseases complicated by chronic viral infections, viral reactivation has been reported after the use of some drugs, such as corticosteroid therapy and immune checkpoint inhibitors (ICIs) for more than 2 weeks [464], and viral reactivation significantly increases the risk of acute liver failure and death [465]. It is worthwhile to investigate when and how MSCs can be applied to strike a balance between antiviral immunity and mitigation of liver pathological damage.

### MSC for alcoholic hepatitis

The most commonly used clinical corticosteroids improve short-term survival in patients with severe AH, but do not provide long-term survival benefits [466]. In addition, approximately one quarter of patients with severe AH did not respond to corticosteroid therapy [467], and liver transplantation is still the most effective treatment for patients with decompensated AH [466]. Given the important role of immune-mediated liver injury in the development of AH, the effectiveness of the corresponding immunotherapeutic strategies has been evaluated (Table 1). However, TNF agents, growth factors and antioxidants have been shown not to show the expected efficacy in clinical trials for the treatment of severe AH [468, 469]. This may be partly attributed to the fact that the inhibition of inflammation also compromises the beneficial effects of inflammation in promoting tissue repair and resistance to microbial infection. The endogenous liver regeneration inducing, immunomodulatory and attenuating liver fibrosis properties of MSCs allow it to play an active role in AH treatment [470, 471]. Recently, several clinical trials on the efficacy of BM-MSCs transplantation in patients with alcoholic cirrhosis have shown that MSCs are effective in reducing the extent of cirrhosis and improving liver function in patients [472–475]. We will focus on the immunomodulatory role of MSCs in AH treatment (Fig. 6d).

Activated recruitment of macrophages and massive infiltration of neutrophils are prominent features of hepatic immunopathology in AH. It has been demonstrated that MSCs can reduce hepatic steatosis and liver injury by inhibiting neutrophil and macrophage infiltration [476]. In addition, a study showed that BM-MSCs injected into AH mice reduce liver injury by secreting the anti-inflammatory factor TSG-6 [477], which may be related to the inhibition of STAT3 activation by TSG-6, thereby inhibiting hepatic oxidative stress and inducing hepatic M2 macrophages polarization in AH mice [478]. Macrophages, however, also have a beneficial aspect of promoting tissue repair. One study investigated the impact of autologous bone marrow stem cells transplantation (SCT) in AH, and patients receiving SCT exhibited a more significant expansion of CD68 + pro-inflammatory hepatic macrophages compared to standard treatment, along with an upregulation of the expression of a gene involved in the regenerative pathway (SPINK1 mRNA) [479]. It has also been found that administration of TLR3 pre-activated BM-MSCs to enhance their immunotherapeutic effects effectively suppressed early intrinsic immune cells NKB cells, reduced IL-18 levels, and improved liver and intestinal injury. This may be associated with reduced activation of NK cells and innate lymphoid 1 cells (ILC1) [480]. Considering the alcohol-mediated disruption of the intestinal barrier and the consequent activation and recruitment of liver immune cells as the initiating link in AH, the strategy of combining probiotics to repair the intestinal mucosal barrier is expected to further enhance the therapeutic effect of MSCs. Lactobacillus rhamnosus GG supernatant (LGG-s) is known to play a beneficial role in alcohol-induced liver injury by improving intestinal barrier function [481]. In a combined study of LGG-s and BM-MSCs, it was found that their synergistic effect on the treatment of AH was specifically manifested as modulating inflammation, accelerating autophagy and reducing the number of alcohol-induced natural killer B (NKB) cells and Tfh cells through PI3k/NF-kB and PI3K/mTOR pathways, and ultimately effectively reducing liver tissue injury and hepatic lipid accumulation [482].

The centrality of immune-mediated liver injury in the pathophysiology of AH has been widely recognized. The treatment of severe AH remains challenging, and controlling inflammation is equally important as controlling infection, but there is a conflict. MSCs seem to achieve better efficacy between suppressing inflammation, controlling infection, and promoting tissue repair, but their specific regulatory mechanisms remain obscure. Clinical studies of MSCs in patients with alcoholic cirrhosis, have shown their effectiveness, but high-quality clinical studies on their treatment of AH, especially severe AH, are lacking. Research is urgently needed to bring benefit to patients with this high mortality disease.

### MSC for NASH

Currently, there is no FDA-approved treatment for NASH. Monotherapies against NASH appear to be challenging, and many targeted therapeutics that were considered promising have been deemed ineffective in recent clinical trials for alleviating the endpoint outcomes of NASH, particularly fibrosis [10, 338]. The strategy of reducing cell death by inhibiting the caspase pathway also brings us important lessons [483]. Although inhibition of caspases may reduce serum ALT in the short term, in the long term, it may drive cells to other cell death mechanisms, leading to more severe liver fibrosis [484].

MSCs have a positive impact on body weight, glucose and lipid metabolic balance, NAFLD and systemic inflammation in obesity treatment[485]. Marcelo et al. found that MSCs intravenously administered to C57BL / 6 mice fed a high-fat diet for a long time did not reverse obesity and metabolic syndrome, but prevented the transition from steatosis to NASH in obese mice with metabolic syndrome [486]. MSCs improved lipid metabolism, insulin resistance, and mitochondrial oxidative stress in NAFLD mice, thereby reducing liver inflammation and fibrotic features [487, 488]. Furthermore, MSCs treatment reversed gut microbiome and metabolome disorders in NASH models, suggesting that MSCs can exert therapeutic effects by affecting the intestinal flora [489]. Indeed, MSCs-exosomes also exhibit therapeutic effects. Treatment of NAFLD rats with human umbilical cord MSCs-exosomes (huMSCs-exos) revealed that miR-627-5p in exosomes could improve NAFLD progression by improving glucose and lipid metabolism and reducing liver injury [490].

The hepatic immune imbalance of NASH triggers inflammation, liver injury and induction of subsequent fibrosis, and studies using immunomodulatory effects of MSCs to correct the imbalance of the immune inflammatory response in NASH for the treatment of the disease are being attempted (Fig. 6e). In a mouse model of NASH, treatment with MSCs improved liver function and morphology and ameliorated liver fibrosis and inflammation. These observed effects may be attributed to the downregulation of pro-inflammatory and pro-fibrotic genes [491]. By developing a NASH model for rapid accumulation of fibrosis, both reduced serum alanine aminotransferase levels and inflammatory markers in model mice following the use of human MSCs and their small extracellular vesicles (sEVs), while liver fibrosis improved and a significant increase in anti-inflammatory macrophages was observed in the livers of mice [492]. The above studies suggest that MSCs can play a therapeutic role in NASH by modulating macrophage phenotypic transformation and thus suppressing inflammation levels. The therapeutic effect of adipose-derived MSCs on obesity-related complications (e.g., NAFLD, CVD, and renal diseases) in animal models of diet-induced obesity derives from the attenuation of inflammatory cytokines such as TNF-α and IL-6 [493]. Similarly, in a high-fat diet (HFD)-induced NASH model, adipose-derived MSCs-EVs significantly reduced the number of Kupffer cells in rat liver and decreased their expression of inflammatory cytokines TNF-α, IL-1β, and IL-6, as well as the secretion of TGF-β. Importantly, treatment with exosomes significantly alleviated liver fibrosis as well as the activation of HSCs [494]. The inhibition of TLR4-mediated signaling in Kupffer cells by adipose-derived MSC-EVs was also confirmed by in vitro assays [494].

Furthermore, after MSCs treatment, MCD diet-induced pathological features of NASH in mice were attenuated, as evidenced by weight loss, hepatic steatosis, hepatocyte expansion, and reduced liver inflammation and fibrosis. Further analysis suggested that a possible mechanism of this MSCs-mediated immunomodulation is the suppression of activation of CD4 + T cells secreting IFN-γ and IL-6 [495]. Another study showed that bone marrow-derived MSCs could attenuate hepatic steatosis, inflammation and fibrosis in NAFLD model mice by suppressing the activation capacity of CD4 + T cells [496].

Preclinical findings suggest that the observed pathological alterations in NASH appear to be related to at least the combined effects of donor MSCs, however, studies on their relevance are limited especially with respect to immune regulation. With the study of NASH-related immunopathogenesis, especially the elucidation of the role of adaptive immunity in the pathology of NASH and the development of NASH-HCC, it will contribute to the development of immunotherapies, including MSCs, to treat NASH and prevent the development of NASH-HCC.

### MSC for liver cirrhosis

Animal studies have shown that the use of MSCs can effectively and safely improve the pathological features of liver fibrosis/cirrhosis and liver function [497, 498]. Early clinical trials also further evaluated the promising therapeutic effects of transplanted MSCs in patients with liver fibrosis [499–505], focusing on improvements in disease scores and indicators of liver function, but with poor evidence of histological improvements. Therefore, preclinical and clinical trials are ongoing to determine the therapeutic potential and safety of MSCs-based therapy in liver diseases (Table 2).

Many studies have described the role of MSCs in cirrhosis, including downregulation of pro-inflammatory and fibrotic cytokine activity, secretion of anti-inflammatory and anti-fibrotic molecules, stimulation of hepatocyte proliferation, inhibition of HSCs cell activation and promotion of collagen degradation through secretion of matrix metalloproteinases (MMPs) [506–511]. MSCs themselves or through paracrine secretion can act directly on HSCs to reduce fibrosis [512–515]. Importantly, MSCs can effectively inhibit NK cells thereby attenuating their mediation of liver injury in diseases such as cirrhosis [516]. Specifically, IDO, TGF-β and PGE2 are the key mediators of NK cells inhibition by MSCs. This inhibition is associated with a dramatic decrease in the expression of NK cells surface receptors CD69, NKp30, NKp44 and NKG2D [516–518]. In addition, MSCs can exert antifibrotic effects by inhibiting the activation of HSCs either directly or indirectly through modulation of immune cells [13] (Fig. 6a).

Some researchers have suggested that MSCs injected into the body are mostly trapped in the lungs, but these MSCs can provide signals to liver macrophages as a way to reduce liver fibrosis [519]. MSCs are dependent on IL-6 and PGE2, among others, to promote the polarization of macrophages toward the anti-inflammatory M2 phenotype [520, 521]. Studies have shown that the remission of liver fibrosis after huMSCs infusion is attributed to the conversion of M1 macrophages to M2 macrophages, with M2 secreting IL-10 and subsequently increasing M1 macrophage apoptosis [522]. MSCs were recently reported to induce changes in the cytokine profile of macrophages and promote resolution of fibrosis [523, 524]. Activation of M2 macrophages and their MMP-13 secretion were significantly increased after transplantation of BM-MSCs, whereas activation of M1 macrophages was suppressed in liver tissue. This was accompanied by an increase in IL-10 gene expression and a decrease in IL-12b, IFN-γ, TNF-α and IL-6 gene expression [523].

MSCs could also promote mobilization of Kupffer cells in vitro and in vivo and induce M1 to M2 conversion by increasing IL-4 and IL-10 secretion, thereby alleviating dimethylnitrosamine (DMN)-induced liver fibrosis [522]. Similarly, bone marrow MSCs transplantation can alleviate liver fibrosis by promoting the phenotypic shift of monocytes from the pre-fibrotic Ly6C^hi^ subpopulation to the restorative Ly6C^low^ subpopulation through paracrine secretion of IL-4 and IL-10 [525]. BM-MSCs produce a large number of apoptotic bodies in the fibrotic liver 72 h after transplantation. Ly6C^low^ macrophages engulf most apoptotic bodies after robust triggering of MMP-12 expression via the PtdSer-MerTK-ERK signaling pathway to alleviate fibrosis [525]. A study used a combination of two cell types, MSCs and colony-stimulating factor 1-induced bone marrow-derived macrophages (id-BMMs), for treatment [524]. On the one hand id-BMMs promoted the migration of host macrophages and neutrophils to the damaged liver, on the other hand ex vivo assays confirmed that MSCs made id-BMMs differentiate into M2 macrophages with high phagocytic activity and high expression of MMP-13, both of which effectively improved liver fibrosis and regeneration through the increase of antifibrotic and pro-regenerative factors [524].

MSCs therapeutic effects are mediated, at least in part, through the regulation of Treg / Th17 cells balance. Studies in mouse models of liver fibrosis have shown that MSCs promotes the expansion of foxp3 + Treg cells and inhibits the proliferation of Th17 cells in an IDO-dependent manner and eventually led to attenuation of liver fibrosis [526]. In another study, transplantation of MSCs was effective in improving liver function in patients with HBV-related cirrhosis, and the increase in hepatic Treg cells and decrease in Th17 cells after MSCs transplantation led to an increase in the Treg/Th17 ratio [527]. IL-17A and its pro-fibrotic effects in the liver are the most studied [528]. IL-17A-producing cells Th17 cells are diverse and can be derived from both Th17 cells, but also from neutrophils, NKT cells, and some innate T cells subsets [529]. In a CCL4-induced rat liver fibrosis model [808], BM-MSCs treatment reduced IL-17, IL-2 and IL-6 serum proteins and downregulated IL-17A and IL-17RA proteins in liver tissues [530]. Further studies showed that BM-MSCs could play a protective role in liver fibrosis treatment by affecting the IL-6/STAT-3 signaling pathway and downregulating IL-17A [530].

MSCs-based cell-free therapy, which is also used in liver fibrosis, helps to circumvent the limitations of cell therapy. In TAA-induced chronic liver injury in rats, MSC-EVs immunomodulatory activity was comparable to that of their parent cells, significantly inhibited the proliferation of peripheral blood mononuclear cells (PBMCs), and resulted in reduced levels of fibrosis and collagen density, necrosis, and inflammation [531]. By cytokine assay and genetic analysis, MSCs significantly increased the secretion of anti-inflammatory factors and decreased the secretion of IFN-γ; and were accompanied by upregulation of collagenase as well as anti-apoptotic genes [531]. In addition, a study delivered TSG-6, a major anti-fibrotic cytokine of MSCs, to the liver via calcium phosphate nanoparticles with the aim of avoiding possible adverse effects of MSCs. The results showed that TSG-6 delivery effectively induced macrophage polarization toward the M2 phenotype and upregulated MMP-12 expression in macrophages, effectively alleviating liver fibrosis [532].

The core of MSCs in the treatment of cirrhosis is the improvement of fibrosis, and MSCs have been shown to reduce the activation of MSCs either directly by targeting HSCs or indirectly through immunomodulation, but the mechanisms involved remain to be deciphered. Clinical studies using MSCs for cirrhosis have been conducted extensively and have confirmed their effectiveness in improving liver function, but their ability to reduce fibrosis still needs to be confirmed in large scale and stratified studies in different chronic liver disease settings. These efforts will clear the way for the large-scale use of MSCs in clinical treatment in the near future.

## MSCs for immunocompromised liver diseases

### MSCs for CVH

The continued development of direct antiviral agents (DAAs) has addressed most of the challenges in the field of chronic HCV treatment and has been so successful in the antiviral treatment of chronic HCV that HCV infection is considered curable [449]. However, some patients remain difficult to cure. Compared to HCV, the dilemma of HBV antiviral treatment still exists. The field generally recognizes the importance of addressing the persistence of covalently closed-loop cccDNA in cells and the high antigenic load (especially hepatitis B surface antigen). However, HBV is present in cells as cccDNA and cannot be eliminated with antiviral drugs [533]. In addition, recovery of the immune response after DAAs therapy is currently under debate, with certain immune features being rejuvenated but some signs of immune exhaustion likely to persist [534–537]. Therefore, treatment of CVH requires restoration of a low antiviral immune response [538], clearance of infected hepatocytes and prevention of reactivation of residual virus (Table 1).

The immunosuppressive activity of MSCs has raised safety concerns regarding primary viral infections and increased risk of viral reactivation. However, MSCs can promote the proliferation of virus-specific immune cells and their antiviral exertion [20, 21]. For example, using the SIV model of AIDS, some researchers have recently found that MSCs can restore antiviral immunity by reconstituting damaged lymphoid follicles, as evidenced by robust regeneration of germinal centers, as well as restoration of follicular B cells and Tfh cells leading to increased levels of anti-SIV antibodies and SIV-specific CD8 + T cells, resulting in viral reduction [539]. Similarly, a phase II randomized, double-blinded, multicenter, placebo- controlled, dose-determination trial in 72 immune nonresponder (INR) patients with chronic HIV-1 infection showed that treatment with huMSCs was safe, with a significant increase in CD4 + T cells counts in HIV-infected patients after 48 weeks of treatment, although a larger cohort studies are needed to further confirm the immune reconstitution efficacy of MSCs [540].

MSCs have been shown to be effective in the treatment of HBV [460, 527, 541–553] and HCV [554–558] related liver failure and cirrhosis. These studies validated the effectiveness of MSCs in improving liver function and prognosis in patients with CVH. Of course, as BM-MSCs allow HBV infection, they may become a viral reservoir after administration. However, research found that adipose-derived MSCs were insensitive to HBV [544] and may be a more suitable source for HBV-related liver diseases. In addition, recent studies have shown that the efficacy of MSCs in the treatment of HBV-associated acute chronic liver failure and cirrhosis varies depending on the age of the patient [559]. In summary, prior to the clinical use of MSCs, assessment of patient's age and further study of the long-term effects of MSCs therapy in HBV-infected patients from different sources are necessary to determine the safety and efficacy of their therapeutic use.

**Fig. 7:**
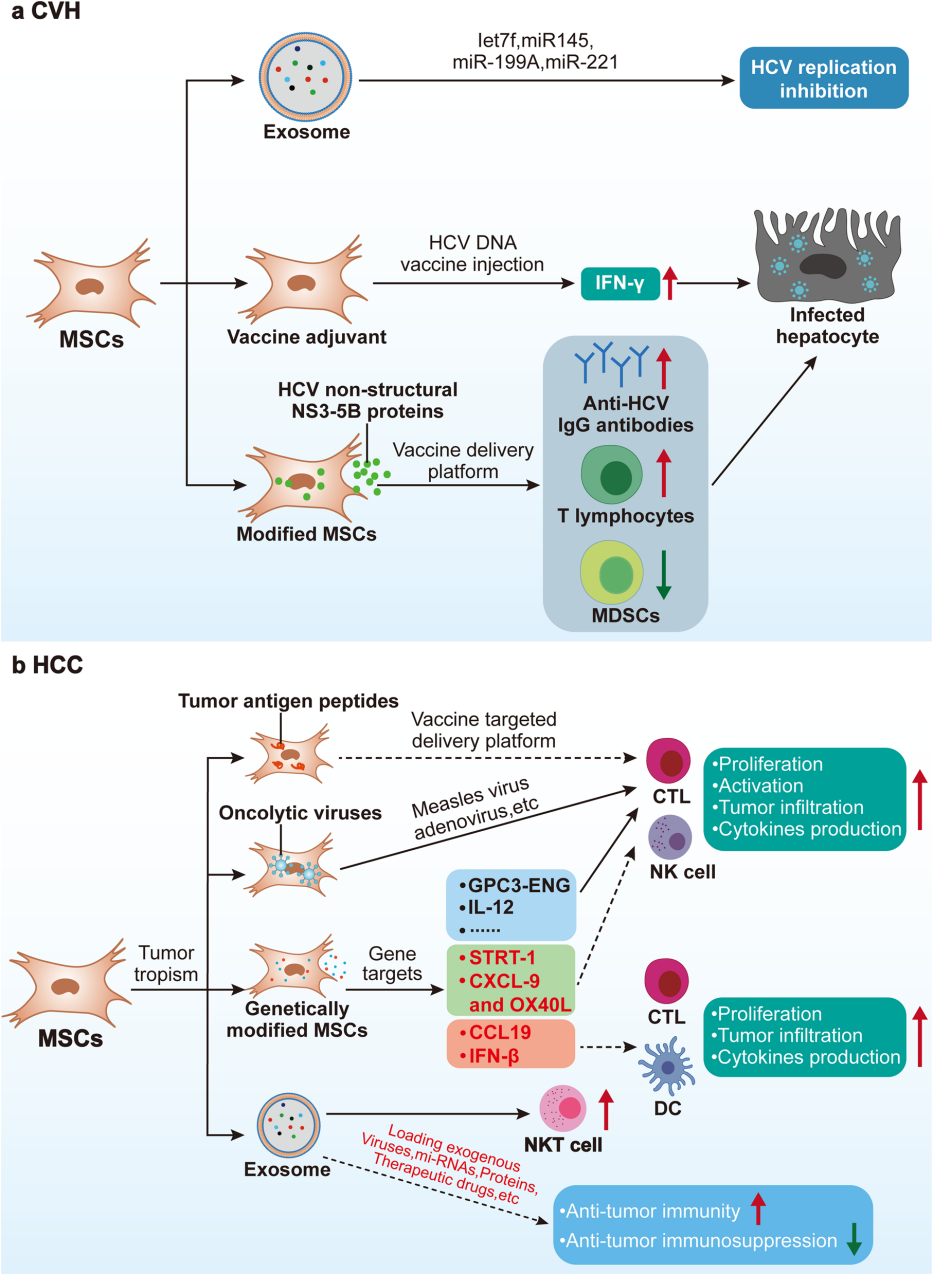
Immunomodulation and immunotherapeutic strategies of MSCs in liver diseases. Due to the immunomodulatory plasticity of MSCs and their ability to deliver immunomodulators after modification, MSCs can activate or enhance antiviral and antitumor immune responses in immunocompromised liver diseases including (**a**) CVH and (**b**) HCC, resulting in clearance of viral and tumor cells. The dashed arrows indicate potential immunomodulatory mechanisms or immunotherapeutic strategies

MSCs can be used as adjuvants for antiviral vaccines and, in addition, MSCs or their exosomes modified by genetic modification and other methods can be used as a delivery vehicle for antiviral vaccines (Fig. 7a). In a study, mouse MSCs simultaneously expressing five non-structural HCV proteins (NS3-NS5B) modified triggered strong phagocytic activity, enhanced T lymphocyte proliferation, anti-HCV non-structural proteins (NS3-NS5B) IgG antibodies production and type I and II interferon production [560]. This antiviral immune effect produced by modified MSCs is thought to be related to the inflammatory cytokine IL-6 affecting the immunomodulatory effects of MSCs and the reduction of MDSCs [560]. A study by the same research group further showed that bone marrow MSCs also exhibit adjuvant properties in hepatitis C DNA vaccination [561]. When MSCs were administered prior to hepatitis C DNA vaccination, low levels of cytokines in mice allowed MSC type 1 to effectively enhance the immunostimulatory activity of mice against DNA vaccine. In contrast, high levels of pro-inflammatory cytokines detected after DNA vaccination promoted the conversion of MSC type 2, leading to suppression of antiviral immunity [561].

MSCs can also effectively inhibit the replication of HCV and exhibit antiviral effects. The huMSC-Exos contain four microRNAs (miRNAs), including let-7f, miR-145, miR-199a, and miR-221, which are capable of inhibiting HCV RNA replication. It was found that exosomes may exert antiviral effects by transporting these microRNAs with HCV-RNA binding sites to infected hepatocytes [556]. In addition, huMSC-Exos shows synergistic effects with IFN-α or telaprevir in the inhibition of HCV replication and is considered as a new adjuvant therapeutic agent for the treatment of HCV patients [556].

Despite the risk of viral transmission as well as immunosuppressive properties, studies have demonstrated the safety and efficacy of MSCs in viral liver diseases. The use of MSCs-exos and the loading of immunotherapeutic agents by biomodified MSCs and their extracellular vesicles are expected to avoid these risks while enhancing hepatic antiviral immunity. However, it should be noted that the additional liver damage associated with restored/enhanced immunity may affect its immunotherapeutic efficacy [108, 534, 562], which also applies and needs to be carefully evaluated in MSCs.

### MSCs for HCC

As mentioned earlier, chronic liver disease induces a tumor microenvironment that promotes tumor development and metastasis through a variety of inflammatory and non-inflammatory precancerous factors, in addition to the formation of tumor cells and tumor tissue that can further consolidate the immunosuppressive microenvironment that promotes their development (Fig. 4c). To reverse the tumor-induced immunosuppressive microenvironment, a number of immunotherapeutic approaches have targeted the aforementioned critical steps [563] (Table 1). ICIs such as PD-1/PD-L1 have been used in immunotherapy regimens for HCC patients with impressive success [74, 564]. However, immune checkpoint-based immunotherapies still have a long way to go in the face of low response rates and appear to exhibit negative effects in NASH-HCC that impair immune surveillance and promote tumorigenesis[199]. And PD-1 inhibitor treatment may lead to Treg cells expansion and further suppression of antitumor immunity, causing tumor hyper-progressive disease (HPD) [565]. Recent studies have shown that HPD can occur in patients with advanced hepatocellular carcinoma treated with anti-PD-1 antibodies, suggesting that ICIs may even be harmful in this setting [566]. Moreover, the occurrence and progression of HCC involves various mechanisms and microenvironmental changes, and there is a fundamental question regarding targeted drug therapy (MTA), i.e., whether a targeted therapy is effective. In fact, most target drug candidates have not shown the expected therapeutic effect in phase II or phase III clinical trials [567]. Combining two or more targeted agents for treatment to exploit synergistic effects may be a more effective strategy for the treatment of HCC [74].

Although in vitro cellular experiments have shown that MSCs and their exosomes effectively inhibit the proliferation of hepatocellular carcinoma cell lines [568–570], which involves multiple signaling pathways [571], some evidence suggests that MSCs also promote the proliferation and progression of HCC [572–576]. The specific regulatory mechanisms regarding the promotion or suppression of tumors by MSCs remain unclear. Differences in the tumor-promoting or tumor-suppressing effects of MSCs depend, at least in part, on the high or low level of inflammation in the microenvironment [18, 19]. In addition, the ability of MSCs to exert anti-tumor immunity may vary depending on their tissue origin. MSCs from reproductive tissu es (e.g., uterus, umbilical cord, or placenta) have more potent antitumor effects and tropism towards tumor tissue [577]. Besides, the composition of the contents of MSC-EVs varies with the MSCs source and ultimately has different effects on tumor fate [578].

Based on the conflicting results of direct use of MSCs in the treatment of HCC and the risk of tumor promotion, current strategies for MSCs in the treatment of HCC mainly include genetically modified MSCs [579, 580], and as vectors for the delivery of oncolytic viruses [581, 582], bioactive proteins [583], antitumor drugs [447, 584] and suicide genes [585, 586], especially their derived extracellular vesicular EVs [587, 588]. The aim of the above strategies is to enable MSCs to exert an efficient anti-tumor capacity while minimizing the pro-tumor risk. For example, IFN-β-modified MSCs can effectively inhibit HCC proliferation in vitro and in vivo by blocking AKT/FOXO3a signaling in HCC cells [580]. Importantly, exploiting the immunomodulatory capacity of MSCs and their advantages as alive cells carriers to enhance antitumor immune responses is a more promising strategy [22, 589], and we will highlight the progress of MSCs research in this regard (Fig. 7b).

#### Oncolytic virus-MSCs

MSCs loaded with tumor lytic viruses activate and enhance the anti-tumor immune response mainly by inducing immunogenic cell death (ICD) and releasing tumor antigens (TAAs) [590]. MSCs using tumor tropism to deliver measles virus to tumor microenvironment significantly inhibited the growth of hepatocellular carcinoma in mice by administration [591]. In another study, adenoviral vectors carrying anti-CD3scfv were firstly constructed with the aim of infecting and modifying HCC cells to highly express anti-CD3scfv, and secondly engineered MSCs were used to load this adenoviral vector to efficiently target HCC tissues. The results showed that MSCs successfully targeted tumor tissues and HCC cells highly expressing anti-CD3scfv effectively activated CTLs, thus inhibiting the growth of HCC [592]. In mouse models of renal adenocarcinoma and melanoma, treatment with lysovirus dlE102 (OAd)-MSCs reduced tumor volume by 50%, which was associated with increased tumor tissue infiltration by TAMs, NK cells and TILs and a reduced proportion of PD-1 + TILs [593]. This suggests that this MSC treatment system is effective in inducing activation of the human immune system, thus exerting anti-tumor effects.

#### Cancer vaccine-MSCs

MSCs and their derived exosomes can be genetically modified or otherwise loaded with cargo to become antigen expression vectors or antigen libraries for use as cancer vaccine delivery platforms [594]. In a phase I/II clinical trial, mature DCs enriched with HCC antigens such as α-FP, glypican-3 (GPC-3) were given as a cancer vaccine to patients with advanced HCC and induced an effective T-cells response as evidenced by high levels of IL-12 and IFN-γ production, with radiographic regression and disease stabilization in 13.3% and 60% of patients, respectively [595]. Predictably, MSCs could also carry HCC antigens or related antigenic peptides as cancer vaccine delivery vehicles because of their tumor-targeting and antigen-presentation capabilities. The strategy of MSCs as cancer vaccine targeted delivery and presentation of antigenic peptides to T cells has shown initial success in tumor models such as mouse lymphoma as well as melanoma [596, 597], although the lack of HCC highly immunogenic tumor antigens and neoantigens may pose some challenges for HCC vaccine development [598].

#### Genetically modified MSCs

Genetically modified MSCs coupled with the ability of these cells to migrate to tumor sites can be used as an effective tool to boost the body's anti-tumor immunity. Bone marrow MSCs have been explored as carriers for the delivery of bispecific T cell-junction antibodies, which bind tumor antigens and specific T lymphocytes [599]. Szoor and colleagues used MSCs that expressed bispecific T-cell splicing agents (GPC3-ENG) targeting Glypican 3 (GPC3) and CD3, to direct GPC3-specific CD4 + T helper cells and CD8 + CTLs towards the GPC3-expressing HCC cells. Increased IFN-γ production by GPC3-specific CD4 + T cells and enhanced activation and amplification of GPC3-specific CTLs in vitro and in vivo, resulting in CTLs-dependent effective killing of GPC3-expressing HCC cells [599]. Furthermore, IL-12 genetically engineered MSCs can preferentially appear at primary tumor sites in mice and at spontaneous metastasis sites pre-established by subcutaneous injection of hepatocellular carcinoma cells, representing its tumor suppressive effect [600]. Another study using radiation and MSCs expressing IL-12 increased the inhibition of hepatocellular carcinoma, including inhibition of lung metastasis and improved survival [601]. Further studies found increased expression of IL-12 in tumor cells and caused proliferation of CD8 + T cells and NK cells [601].

Using a liver metastasis model of colorectal cancer, SIRT-1 overexpressing MSCs were recently shown to exert antitumor activity by increasing the number of CD8 + T cells [602]. In other solid tumors, MSCs overexpressing SIRT-1 can induce NK cell recruitment at breast and prostate cancer tumor sites in vivo and promote their antitumor activity by enhancing IFN-γ secretion of NK cells [603, 604]. Pan et al. overexpressed the T/NK cells-targeting chemokine CXCL9 and the immunostimulatory factor OX40 ligand (OX40L) in MSCs by lentiviral transfection, which increased the proportion of infiltrating CD8 + T and NK cells in colon cancer tumors, the production of antitumor cytokines and cytolytic proteins in the tumor microenvironment, thus overcoming the systemic toxicity of therapeutic agents and the problem of low lymphocyte infiltration in solid tumors [605]. In another study, chemokine ligand 19 (CCL19) was generated by genetic modification, successfully attracted CCR7 + DCs and CD8 + T cells that secrete IFN-γ, and effectively inhibited colon cancer growth. And the anti-tumor phase effect was better in the case of combined anti-PD-L1 antibody treatment [606]. In gliomas, the persistence of secreted IFN-β around gliomas after transplantation of MSCs infected with lentiviral overexpression of IFN-β and ferritin heavy chain (FTH) led to a significant infiltration of Batf3 + DCs and CD8 + T cells in gliomas and was able to effectively treat malignant gliomas [607]. These potential gene modification targets for MSCs have not been investigated in HCC, but their ability to enhance anti-tumor immunity in solid tumors is attractive and deserves further validation in the treatment of HCC.

#### MSCs-exos

Research confirms that adipose-derived MSC-exos can inhibit HCC growth in rat models, possibly associated with increased numbers of circulating and intra-tumor NKT cells [608]. In addition, MSC-exos can transfect specific miRNAs to effectively inhibit HCC development [609, 610]. In other solid tumor models, miR-182 released from huMSCs-exos killed clear cell renal cell carcinoma (ccRCC) by suppressing VEGFA expression promoting increased numbers of DCs, NKT cells and CD8 + T cells as well as enhancing the sensitivity of tumor cells to NKT cells [611]. A recent study utilized an exosome-based dual delivery biological system for the treatment of pancreatic cancer [612]. The delivery system consisted of BM-MSC-exos loaded with galectin-9 siRNA by electroporation, and surface-modified oxaliplatin (OXA) prodrug as an ICD trigger. This combination therapy reversed the tumor immunosuppression of M2-like TAMs (M2-TAMs) by disrupting the galectin-9/dectin1 axis and recruited cytotoxic T cells and downregulated Treg cells to stimulate anti-tumor immunity with significant efficacy in cancer therapy [612].

In conclusion, despite the risk associated with tumor promotion, MSCs can effectively activate the hepatic anti-tumor immune response through genetic modification and loading of tumor antigenic peptides, cancer vaccines and other oncology drugs, and show good potential for the treatment of HCC. In addition, MSCs have immunomodulatory functions, superior tumor targeting and the ability to reach inside tumor tissues, which is unmatched by other vectors [22].

## Conclusion and prospect

The treatment of liver diseases cannot be separated from the discussion of the hepatic immune microenvironment, a complex and ever-changing system whose full picture has not been fully revealed, especially in pathological states. Many immunotherapies have been developed and used to treat liver diseases, but the complexity of the hepatic immune microenvironment makes single-target therapy unsatisfactory and combination of drugs may be a more effective therapeutic strategy. In addition, more advanced and comprehensive multi-omics techniques are being used to dissect the immune interaction landscape in the liver to identify potential therapeutic targets for specific diseases.

Clinical trials of MSCs for the treatment of liver diseases have been extensively conducted, with most studies focusing on advanced liver disease and cirrhosis to verify safety and efficacy. Their therapeutic effects have been interpreted as a combination of immunomodulatory, regeneration-promoting and anti-fibrotic capabilities. From the perspective of immunopathogenesis of liver disease, the immunomodulatory capacity of MSCs needs to be carefully investigated to assess whether the combined and plastic immunomodulatory capacity of MSCs has significant advantages, especially if it can overcome the poor effect of single immune target therapy. This is crucial for the further application of MSCs in the treatment of liver diseases.

Still, the immunomodulatory ability of MSCs in viral infections and tumors is controversial and requires further studies to clarify as well as strict quality control during clinical use to avoid their potential risks. Compared to conventional unmanipulated MSCs, the overall efficacy and/or organ homing of MSCs has been improved in recent years by genetically engineering MSCs [613] and by using MSCs-derived exosomes as drug carriers [614]. Exosomes, a cell-free therapy, also avoid the potential risk of hypermigration and carcinogenesis of MSCs [614]. Therefore, MSCs are promising for the treatment of liver diseases.

Furthermore, the assessment of the therapeutic efficacy of MSCs does not include their effectiveness in halting the progression of chronic liver disease to cirrhosis and HCC. Considering the refractory nature of end-stage liver disease and HCC, the high mortality rate and the need for clinical control, it is necessary to evaluate the role of MSCs in this regard in the future. In addition, there are differences in the immunomodulatory mechanisms and focus of MSCs in acute and chronic viral hepatitis, and future clinical and preclinical studies should pay attention to the distinction in the selection of study subjects. Immune dysfunction in end-stage liver diseases such as advanced cirrhosis, liver failure, and HCC is often characterized by the coexistence of inflammation and immune deficiency, and different etiologies may lead to different immune landscapes in end-stage liver diseases. Therefore, further etiologically stratified, randomized, high-level clinical studies are needed to improve the reliability of the clinical efficacy of MSCs in order to establish MSCs therapy as a clinical option for these liver diseases. The efficacy of MSCs in combination with other immunotherapeutic agents for the treatment of liver disease has not been fully evaluated. In addition, further research on the optimal source, the best route of administration, sufficient number of MSCs and prolonged survival of transplanted MSCs is needed to improve the efficacy of MSCs therapy.

In conclusion, MSCs are promising immunotherapeutic approaches for liver diseases, and the specific cellular and molecular mechanisms of MSCs immunomodulation will be further clarified as the immunopathogenesis of liver diseases is further investigated. MSCs, together with other immunotherapeutic agents, are expected to advance the field of immunotherapy for liver diseases.

## Data Availability

Not applicable.

## Abbreviations

ADCC: Antibody-dependent cell-mediated cytotoxicity
Breg: Regulatory B cell
BAFF: B cells activation factor
CXCL: Chemokine (CXC motif) ligand
CCL: Chemokine (C–C motif) ligand
cccDNA: Covalently closed circular DNA
CTLA-4: Cytotoxic T lymphocyte-associated antigen-4
EVs: Extracellular vesicles
FasL: Fas ligand
FGF: Fibroblast growth factor
G-CSF: Granulocyte colony-stimulating factor
HLA-G: Human leukocyte antigen-G
HGF: Hepatocyte growth factor
HO-1: Heme oxygenase-1
5-HT: 5-Hydroxytryptamine
IDO: Indoleamine 2, 3-dioxygenase
iNOS: Inducible nitric oxide synthase
LPS: Lipopolysaccharide
IFNRs: Interferon receptors
ICRs: Immune checkpoint receptors
IL: Interleukin
LAG-3: Lymphocyte activation gene-3
M1: Macrophage type 1
M2: Macrophage type 2
MDSC: Myeloid‐derived suppressor cells
miR: Micro ribonucleic acid
MerTK: Macrophage c-mer tyrosine kinase
NLRP3: The NACHT, LRR, and PYD domains-containing protein 3
NTCP: Sodium-taurocholate co-transporting polypeptide
OSEs: Epitopes of oxidative stressors
OPN: Osteopontin
PD-1: Programmed cell death protein-1
PD-L1: Programmed death ligand-1
PDGF: Platelet derived growth factor
PGE2: Prostaglandin E2
ROS: Reactive oxygen species
Th1: T helper cell, type 1
Th2: T helper cell, type 2
Th17: T helper cell, type 17
Tfh: T follicular helper cells
TNF-α: Tumor necrosis factor-alpha
TLR4: Toll-like receptors 4
TSG-6: Tumor necrosis factor alpha stimulated gene-6
TGF-β: Transformation growth factor-beta
TREM: Triggering receptor expressed on myeloid cells
VEGF: Vascular endothelial growth factor
